# Interplay of Aggregation-Induced Enhanced Emission and Thermally Activated Delayed Fluorescence in Asymmetric Fluorenyl–Benzothiadiazole Derivatives

**DOI:** 10.1021/acsphyschemau.5c00118

**Published:** 2025-12-15

**Authors:** Carolina Vesga-Hernández, Rafael S. Carvalho, Aline M. Santos, Marlin J. P. Peñafiel, Luiz Maqueira, Davi F. Back, Ricardo Q. Aucélio, Fabiano Rodembusch, Flavio Franchello, Edson Laureto, Marco Cremona, Jones Limberger

**Affiliations:** † Department of Chemistry, 28099 Pontifícia Universidade Católica do Rio de Janeiro, Rio de Janeiro, Rio de Janeiro 22451-900, Brazil; ‡ Department of Physics, Pontifícia Universidade Católica do Rio de Janeiro, Rio de Janeiro, Rio de Janeiro 22451-900, Brazil; § Department of Chemistry, 28118 Universidade Federal de Santa Maria, Santa Maria, Rio Grande do Sul 97105-900, Brazil; ∥ Institute of Chemistry, 28124 Universidade Federal do Rio Grande do Sul, Porto Alegre, Rio Grande do Sul 91501-970, Brazil; ⊥ Department of Physics, 37894 State University of Londrina, Londrina, Paraná 86057-970, Brazil

**Keywords:** benzothiadiazole, fluorene, aggregation-induced enhanced emission, thermally activated delayed fluorescence, organic light emitting diode

## Abstract

Combining thermally activated delayed fluorescence (TADF) with aggregation-induced enhanced emission (AIEE) provides an effective strategy to improve solid-state emission in organic materials. Here, we design four fluorenyl–benzothiadiazole (FL-BTD) derivatives bearing additional donor groups, aryloxy (−OAr), aryl (−Ar), iminodibenzyl (−IDB), and phenoxazine (−PXZ), to investigate how molecular structure influences their photophysical properties. FL-BTD-OAr and FL-BTD-IDB display AIEE, with quantum yields that are significantly higher in the solid state (0.70 and 0.30, respectively) than in solution. FL-BTD-IDB also exhibits delayed emission (*t*_d_ = 1.03 μs) and enhanced luminescence under vacuum compared to an O_2_ atmosphere, consistent with TADF. Organic light-emitting diodes (OLEDs) fabricated with these materials show green emission (FL-BTD-Ar and FL-BTD-OAr) or orange emission (FL-BTD-IDB). Device performance trends closely follow the solid-state photophysics. FL-BTD-Ar, subject to partial aggregation quenching, delivers the weakest performance, whereas FL-BTD-OAr benefits from AIEE, resulting in improved brightness and efficiency. The best-performing device is based on FL-BTD-IDB, which combines AIEE and TADF to achieve high brightness (15,000 cd m^–2^) and higher external quantum efficiency (EQE) compared to the analogs.

## Graphical abstract

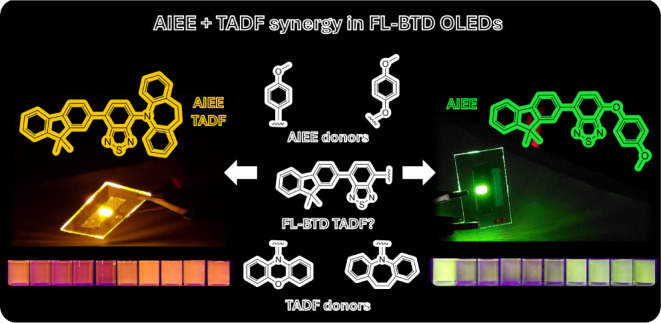

## Introduction

The development of organic molecules that combine thermally activated delayed fluorescence (TADF) and aggregation-induced enhanced emission (AIEE) has attracted increasing interest owing to their potential for efficient solid-state light emission. Molecular systems that integrate these two effects enable more effective exciton utilization while overcoming aggregation-caused quenching (ACQ), a major limitation for organic luminophores in the condensed phase.^1–4^ Rational molecular design is essential for achieving these properties, particularly in donor–acceptor (D-A) and donor–acceptor–donor (D-A-D) architectures. In these systems, electronic and steric modulation can simultaneously promote reverse intersystem crossing (RISC) and suppress detrimental π–π stacking interactions, both of which are critical to realizing TADF and AIEE.^5–7^

Among the electron-accepting cores used in such architectures, 2,1,3-benzothiadiazole (BTD) has emerged as a versatile building block due to its strong electron-withdrawing character, planar structure, and excellent thermal and chemical stability.^8–12^ BTD-based compounds have been widely explored as luminescent materials, benefiting from tunable photophysical properties and modular synthesis.^13–15^ Several BTD derivatives have been reported to display TADF through efficient RISC, particularly in symmetrical D-A-D structures. However, not all donor units typically associated with TADF prove effective when incorporated into BTD framework.^6^ For example, D-A-D BTD derivatives featuring carbazole, diphenylamine, or dimethylacridine donors have been synthesized,^16^ but only the later, BTZ-DMAC, exhibited clear TADF, with emission at 636 nm and a photoluminescence quantum yield (PLQY) of 0.56 in CBP films. TPACNBz, a symmetrical BTD derivative with near-infrared TADF, combines triphenylamine donors with a 5,6-dicyano-substituted BTD core.^17^ Although triphenylamine groups are often linked to AIE/AIEE behavior,^18,19^ this compound suffers pronounced ACQ, as shown by the PLQY drop from 70% in solution to only 21% in film. In contrast, symmetrical BTD derivatives incorporating phenoxazine (PXZ) or iminodibenzyl (IDB) donors, despite their strong electron-donating nature and previous use in TADF systems, do not exhibit delayed emission, with fluorescence that is weak or negligible in both solution and solid state.^20^ In addition, symmetrical D-A-D BTD compounds incorporating non-heteroaromatic donors such as 9,9–dimethyl-9H-fluorene (FL) have been reported to display hot-exciton TADF.^21^ While these BTD-FL systems achieve near unity PLQY in solution, they undergo strong emission quenching in the aggregated state.

AIEE in BTD-based luminophores has also been extensively investigated, particularly through structural modifications that suppress nonradiative decay in the condensed phase. One notable strategy involves aryloxy substituents, which endow BTD derivatives with strong AIEE behavior and up to 30-fold emission enhancement upon aggregation, attributed to the restriction of intramolecular motion by the aryloxy group.^20,22–24^ Similarly, BTDs functionalized with IDB donors display AIE, including viscosity-sensitive fluorescence and enhanced emission in films and powders.^25^ Such motifs have thus proven effective in tuning the solid-state luminescence of BTD derivatives.

Beyond traditional symmetrical D-A-D designs, asymmetric BTD-based systems offer an alternative approach, introducing two distinct donor groups with complementary roles. A representative example is BCZ-BTD-AD, which integrates a 9,9-dimethylacridine donor with a diaryl-carbazole on the BTD core.^26^ This molecule exhibits both delayed fluorescence and enhanced emission in the aggregated state, while its symmetrical counterpart bearing only carbazole groups shows AIEE but lacks TADF.

To further elucidate the interplay between AIEE and TADF in asymmetric BTD-based systems, we designed, synthesized, and characterized four new fluorenyl–benzothiadiazole derivatives: FL-BTD-Ar, FL-BTD-OAr, FL-BTD-PXZ, and FL-BTD-IDB. These molecules share a BTD acceptor core with a dimethyl-fluorenyl unit and a second donor group chosen for its ability to modulate excited-state properties at the complementary BTD position. The choice of donors was guided by previous reports where similar substituents enhanced emission in aggregated states or promoted delayed fluorescence in other BTD derivatives. This modular design enables a systematic investigation of how electronic effects and D–A geometry govern aggregate/solid-state emission and radiative decay efficiency, key parameters for developing efficient emitters in photonic and optoelectronic applications.

## Methods

### General Information

All reagents and solvents were purchased from commercial suppliers and used without further purification unless otherwise specified. Solvents employed in cross-coupling reactions (Buchwald-Hartwig and Suzuki) were deaerated under nitrogen flow. Compounds were characterized by ^1^H and ^13^C NMR spectroscopy (Bruker Avance III HD 400 MHz spectrometer). Detailed NMR characterization data for the novel compounds are provided in Tables Sx and Sy. High-resolution ESI mass spectrometry (ESI-HRMS) operating in positive mode (MICROTOF—Bruker Daltonics), FTIR spectroscopy with the Eco-ATR QuickSnap Sampling Module (Bruker ALPHA II FTIR spectrometer), and, when suitable single-crystal structures were obtained, by single-crystal X-ray diffraction (Bruker D8 Venture Photon 100 diffractometer).

### Synthesis

All reactions were carried out in oven-dried screw-capped Schlenk flasks under nitrogen atmosphere using standard Schlenk techniques. When required, degassed solvents were employed. Intermediates 2, 3, and 4 were prepared as previously reported.^20,27,28^ Full details are provided below.

#### Intermediate 2

4-Bromo-7-(4-methoxyphenyl)­benzo­[*c*]­[1,2,5]­thiadiazole. A mixture of 4,7-dibromobenzo­[*c*]­[1,2,5]­thiadiazole (1) (588 mg, 2.0 mmol), Pd­(OAC)_2_ (44.9 mg, 0.2 mmol), PPh_3_ (104.9 mg, 0.4 mmol), K_2_CO_3_ (553 mg, 4.0 mmol), and 4-methoxyphenylboronic acid (304 mg, 2.0 mmol) in degassed ethanol (5 mL) and toluene (5 mL) was stirred at 75 °C for 24 h. After cooling to room temperature, the solid was washed with ethyl acetate (3 × 5 mL) and the filtrate was concentrated under reduced pressure. The crude product was purified by column chromatography on silica gel using hexane/ethyl acetate as the mobile phase. Yellow solid. 57% yield. ^1^H RMN (400 MHz, CDCl_3_, δ): 7.89 (d, *J* = 7.6 Hz, 1H), 7.88–7.84 (m, 2H), 7.52 (d, *J* = 7.6 Hz, 1H), 7.08–7.05 (m, 2H), 3.89 (s, 3H).

#### Intermediate 3

4-(4-Methoxyphenoxy)-7-bromobenzo­[c]­[1,2,5]­thiadiazole. A mixture of 1 (294 mg, 1.0 mmol,), 4-methoxyphenol (248 mg, 2.0 mmol), K_2_CO_3_ (415 mg, 3.0 mmol,) and DMF (6.5 mL) was stirred at 100 °C for 24 h. After cooling to room temperature, the mixture was diluted with water (10 mL) and extracted with ethyl acetate (3 × 15 mL). The combined organic layers were dried over sodium sulfate, filtered, and concentrated under reduced pressure. The crude product was purified by column chromatography on silica gel using hexane/ethyl acetate as the mobile phase. Yellow solid. mp: 120–122 °C. 54% yield. ^1^H NMR (400 MHz, CDCl_3_, δ): 7.65 (d, *J* = 8.1 Hz, 1H), 7.15–7.09 (m, 2H), 6.99–6.93 (m, 2H), 6.60 (d, *J* = 8.1 Hz, 1H), 3.84 (s, 3H).

#### Intermediate 4

4-Bromo-7-(9,9-dimethyl-9*H*-fluoren-2-yl)-2,1,3-benzothiadiazole. Following a modified procedure from the literature,^28^ compound 1 (150 mg, 0.51 mmol), Pd­(OAC)_2_ (0.56 mg, 2.5 × 10^–3^ mmol), PPh_3_ (1.31 mg, 4.9 × 10^–3^ mmol), K_2_CO_3_ (23.49 mg, 0.17 mmol), 9-dimethyl-9*H*-fluoren-2-yl boronic acid (40.5 mg, 0.17 mmol) in degassed ethanol (1.5 mL) and toluene (1.5 mL) were stirred at 75 °C for 5 h. After cooling to room temperature, the mixture was washed with dichloromethane (3 × 5 mL), concentrated under reduced pressure, and purified by column chromatography on silica gel using hexane/dichloromethane as the mobile phase. Green solid. mp: 126–129 °C. 86% yield. ^1^H NMR (400 MHz, CDCl_3_, δ): 7.97–7.93 (m, 2H), 7.92 (dd, *J* = 7.9, 1.6 Hz, 1H), 7.87 (d, *J* = 7.9 Hz, 1H), 7.82–7.76 (m, 1H), 7.65 (d, *J* = 7.6 Hz, 1H), 7.50–7.45 (m, 1H), 7.41–7.32 (m, 2H), 1.57 (s, 6H).

#### Synthesis of FL-BTD Derivatives via Suzuki Cross-Coupling

Intermediates 2 or 3 (0.16 mmol) were coupled with 9-dimethyl-9H-fluoren-2-yl boronic acid (43 mg, 0.16 mmol) in the presence of Pd­(OAc)_2_ (0.56 mg, 2.5 × 10^–3^ mmol), PPh_3_ (1.31 mg, 4.9 × 10^–3^ mmol), and K_2_CO_3_ (23.49 mg, 0.17 mmol) in degassed ethanol (1.5 mL) and toluene (1.5 mL). The reactions were stirred at 75 °C for 5 h and then cooled to room temperature. The mixtures were washed with ethyl acetate (3 × 5 mL), concentrated under reduced pressure, and purified by column chromatography on silica gel (hexane/ethyl acetate), affording FL-BTD-Ar and FL-BTD-OAr, respectively.

#### FL-BTD-Ar

4-(9,9-Dimethyl-9*H*-fluoren-2-yl)-7-(4-methoxyphenyl)­benzo­[*c*]­[1,2,5]­thiadiazole. Green solid. mp: 189–191 °C. 62% yield. ^1^H NMR (400 MHz, CDCl_3_, δ): 8.03 (d, *J* = 1.1 Hz, 1H), 8.00 (dd, *J* = 7.8, 1.6 Hz, 1H), 7.98–7.94 (m, 2H), 7.89 (d, *J* = 7.8 Hz, 1H), 7.85 (d, *J* = 7.3 Hz, 1H), 7.82–7.78 (m, 1H), 7.77 (d, *J* = 7.3 Hz, 1H), 7.52–7.47 (m, 1H), 7.41–7.33 (m, 2H), 7.14–7.07 (m, 2H), 3.91 (s, 3H), 1.59 (s, 6H). ^13^C NMR (101 MHz, CDCl_3_, δ): 160.0, 154.4, 154.2, 154.1, 139.6, 139.0, 136.6, 133.3, 132.9, 130.6, 130.1, 128.5, 128.2, 127.6, 127.5, 127.2, 123.7, 122.8, 120.4, 120.2, 114.3, 55.6, 47.2, 27.4. FTIR (ATR): ν (cm^–1^) = 3010, 2958, 1607, 1513, 1447, 1343, 1246, 1180, 1028, 831, 737, 513. HRMS (*m*/*z*) calculated for (M + H)^+^: 435.1526, found: 435.1548.

#### FL-BTD-OAr

4-(4-Methoxyphenoxy)-7-(9,9-dimethyl-9*H*-fluoren-2-yl)­benzo­[*c*]­[1,2,5]­thiadiazole: Green solid. mp: 124–126 °C. 95% yield. ^1^H NMR (400 MHz, CDCl_3_, δ): 7.94 (d, *J* = 1.0 Hz, 1H), 7.90 (dd, *J* = 7.9, 1.6 Hz, 1H), 7.85 (d, *J* = 7.9 Hz, 1H), 7.80–7.76 (m, 1H), 7.62 (d, *J* = 7.9 Hz, 1H), 7.49–7.45 (m, 1H), 7.40–7.31 (m, 2H), 7.21–7.16 (m, 2H), 7.01–6.96 (m, 2H), 6.84 (d, *J* = 7.9 Hz, 1H), 3.85 (s, 3H), 1.57 (s, 6H). ^13^C NMR (101 MHz, CDCl_3_, δ): 156.8, 155.0, 154.0, 154.0, 149.9, 148.7, 148.6, 139.1, 138.8, 136.3, 128.7, 128.2, 128.1, 127.4, 127.1, 123.3, 122.7, 121.6, 120.2, 120.1, 115.1, 111.4, 55.7, 47.0, 27.2. FTIR (ATR): ν (cm^–1^) = 3055, 2964, 2925, 1591, 1546, 1509, 1482, 1340, 1248, 1224, 1061, 1026, 897, 834, 815, 735, 505. HRMS (*m*/*z*) calculated for (M + H)^+^: 451.1474, found: 451.1472.

#### Crystal Data

CCDC 2479806 and 2479807 contains the supplementary crystallographic data for this paper. These data can be obtained free of charge from The Cambridge Crystallographic Data Centre via www.ccdc.cam.ac.uk/data_request/cif.

#### Synthesis of FL-BTD Derivatives via Buchwald–Hartwig Amination

Intermediate 4 (100 mg, 0.25 mmol) was reacted with either phenoxazine or iminodibenzyl (0.37 mmol) in the presence of Pd­(OAc)_2_ (2.25 mg, 0.01 mmol), tri*tert*-butylphosphonium tetrafluoroborate (8.7 mg, 0.03 mmol), sodium *tert*-butoxide (36 mg, 0.37 mmol) and dry toluene (2.0 mL). The reactions were stirred at 110 °C for 24 h and cooled to room temperature. The mixtures were diluted with dichloromethane (30 mL) and washed with water. The organic layer was dried over sodium sulfate, filtrated, and concentrated under reduced pressure. The crude products were purified by column chromatography on silica gel using hexane/ethyl acetate as the mobile phase.

#### FL-BTD-IDB

4-(10,11-Dihydro-5*H*-dibenzo­[*b*,*f*]­azepin-5-yl)-7-(9,9-dimethyl-9*H*-fluoren-2-yl)­benzo­[*c*]­[1,2,5]­thiadiazole: Orange solid. mp: 163–164 °C. 39% yield. ^1^H NMR (400 MHz, CDCl_3_, δ): 7.94 (d, *J* = 1.1 Hz, 1H), 7.91 (dd, *J* = 7.9, 1.5 Hz, 1H), 7.84 (d, *J* = 7.9 Hz, 1H), 7.78 (dd, J = 6.5, 1.5 Hz, 1H), 7.60 (d, *J* = 8.0 Hz, 1H), 7.53–7.46 (m, 3H), 7.40–7.31 (m, 2H), 7.31–7.26 (m, 6H), 6.82 (d, *J* = 8.0 Hz, 1H), 3.14 (s, 4H), 1.57 (s, 6H). ^13^C NMR (101 MHz, CDCl_3_, δ): 155.4, 154.0, 153.8, 148.1, 144.9, 139.8, 139.1, 138.3, 137.6, 137.3, 130.4, 129.4, 129.1, 127.8, 127.2, 127.1, 127.0, 126.8, 124.4, 123.0, 122.6, 120.1, 119.9, 110.5, 47.0, 30.9, 27.3. IR (ATR): ν (cm^–1^) 3065, 2979, 2960, 1544, 1482, 1445, 1357, 1273, 825, 760, 735. HRMS (*m*/*z*) calculated for (M + H)^+^: 522.1998, found: 522.2016.

#### FL-BTD-PXZ

10-(7-(9,9-Dimethyl-9*H*-fluoren-2-yl)­benzo­[*c*]­[1,2,5]­thiadiazol-4-yl)-10*H*-phenoxazine: Red solid. mp: 142–144 °C. 79% yield. ^1^H NMR (400 MHz, CDCl_3_, δ): 8.07 (d, *J* = 1.3 Hz, 1H), 8.04 (dd, *J* = 7.9, 1.5 Hz, 1H), 7.96–7.91 (m, 2H), 7.85–7.80 (m, 2H), 7.53–7.48 (m, 1H), 7.44–7.35 (m, 2H), 6.79 (dd, *J* = 7.9, 1.4 Hz, 2H), 6.70 (td, *J* = 7.7, 1.4 Hz, 2H), 6.57 (td, *J* = 7.7, 1.5 Hz, 2H), 5.91 (dd, *J* = 7.9, 1.4 Hz, 2H), 1.61 (s, 6H). ^13^C NMR (101 MHz, CDCl_3_, δ): 155.6, 154.3, 154.2, 153.1, 144.2, 140.2, 138.8, 136.1, 135.8, 133.7, 133.2, 129.5, 128.7, 128.1, 127.9, 127.3, 123.9, 123.4, 122.9, 122.1, 120.5, 120.4, 116.0, 113.5, 47.3, 27.4 IR (ATR): ν (cm^–1^) 3049, 2964, 1735, 1591, 1486, 1330, 1260, 1088, 1043, 1018, 796, 735, 671. HRMS (*m*/*z*) calculated for (M + H)^+^: 510.1635, found: 510.1660.

### Theoretical Calculations

Geometry optimizations and electronic structure calculations were performed using density functional theory (DFT) with B3LYP functional and the 6-31G** basis set,^29^ as implemented in the ORCA 4.2.1 program package.^30^ The commonly used B3LYP (Becke, 3-parameter, Lee–Yang–Parr) exchange-correlation functional approach was employed as indicated for the study of other organic systems.^31,32^ Time-dependent DFT (TD-DFT) was used to evaluate singlet and triplet excited-state energies, singlet–triplet energy gaps (Δ*E*_ST_), and natural transition orbitals (NTOs). Molecular orbitals and electron density distributions were visualized using Avogadro 1.2.0.^33^

### Spectroscopic and Electrochemical Measurements

UV–vis spectra were recorded on a Cary 100 Conc spectrophotometer using 1.0 cm path length quartz cuvettes, a scan rate of 1200 nm min^–1^, and a spectral band-pass of 10 nm. The solution concentrations were around 10^–5^ mol L^–1^. Photoluminescence spectra were obtained on a PerkinElmer LS 55 luminescence spectrometer using a scan rate of 1200 nm min^–1^, a spectral band-pass of 10 nm, and 1.0 cm optical path length quartz cuvettes (four optically clear faces). Reflective neutral density filters (F 2.0, 1.0, and 0.6) were used when necessary to prevent detector saturation. Fluorescence decay times were measured by Time-Correlated Single-Photon Counting (TCSPC) technique using a FluoTime 200 spectrometer (PicoQuant) equipped with a Shimadzu MCP-PMT detector and a 375 nm pulsed diode laser (PicoQuant) as the excitation source. Fluorescence quantum yields in solution were determined by the relative method according to $\phi_{x}=\phi_{\mathrm{St}}\left({\mathrm{Grad}}_{x}/{\mathrm{Grad}}_{\mathrm{St}}\right)\left(\eta_{x}^{2}/\eta_{\mathrm{St}}^{2}\right)$ , where subscripts St and x denote standard and test, respectively, ϕ is the fluorescence quantum yield, Grad is the gradient from the plot of integrated fluorescence intensity vs absorbance and η the solvent refractive index. Solutions were prepared in THF at concentrations between 1.0 × 10^–7^ and 2.5 × 10^–6^ mol L^–1^. Fluorescein sodium salt solutions in NaOH (0.1 mol L^–1^) (PLQY = 0.93) were used as the standard.^34^ Absolute quantum yields in the solid state were measured using Hamamatsu Quantaurus-QY Plus UV-NIR absolute PL quantum yield spectrometer. Aggregate sizes in THF/water mixtures with varying water fractions were determined by dynamic light scattering (DLS) using a Zetasizer NanoSeries model Nano-ZS (Malvern Instruments). Cyclic voltammetry was performed using a potentiostat/galvanostat (μ-AUTOLAB Type III, Metrohm). Measurements employed a glassy carbon working electrode, Ag|AgCl (KCl sat.) reference electrode, and a platinum wire auxiliary electrode, with scan rate of 50 mV s^–1^ under nitrogen at room temperature. The electrolyte solution consisted of 0.04 mol L^–1^ tetrabutylammonium phosphorus hexafluoride (TBAPF_6_) in dichloromethane:acetonitrile (17:3 v/v). Ferrocene was used as internal reference.

### Device Fabrication

Aluminum pellets were purchased from Kurt J. Lesker Company. Indium tin oxide (ITO)-coated glass substrates, molybdenum­(VI) oxide (MoO_3_), *N*,*N*’-bis­(naphthalen-2-yl)-*N*,*N*’-bis­(phenyl)-benzidine (β-NPB), bis-4-(*N*-carbazolyl)­phenyl)­phenylphosphine oxide (BCPO), bis-4-(*N*-carbazolyl)­phenyl)­phenylphosphine oxide (Bepq_2_) and bis-4-(*N*-carbazolyl)­phenyl)­phenylphosphine oxide (Bphen) were purchased from Luminescence Technology Corp. All the materials were used as received. ITO-coated glass substrates were cleaned by sequential ultrasonication in detergent solution, deionized water, acetone, and isopropyl alcohol, followed by UV-ozone treatment at 100 °C for 15 min. The OLED devices were fabricated with the following structures: (i) ITO (150 nm)/MoO_3_ (5 nm)/β-NPB (30 nm)/BCPO:FL-BTD-Ar or FL-BTD-OAr,(30%, 20 nm)/Bphen (40 nm)/LiF (0.5 nm)/Al (70 nm) and (ii) ITO (150 nm)/MoO_3_ (5 nm)/β-NPB (68 nm)/Bebq_2_:FL-BTD-IDB 30% (20 nm)/LiF (0.5 nm)/Al (70 nm). Where MoO_3_ was used as hole injection layer (HIL), β-NPB was used as hole transporting layer (HTL), Bphen was used as electron transporting layer (ETL) (Figure S23). The device structure (i) was inspired by previous studies.^21^ All films were deposited by the thermal evaporation technique in a high vacuum environment (10^–4^ Pa). The deposition system is fully integrated within an MBraun glovebox to prevent exposure of materials and devices to the ambient atmosphere. Deposition rates were 0.4–0.6 Å s^–1^ for organic layers and 1.0–2.0 Å s^–1^ for aluminum. The active area was 3.0 mm^2^. Electrical and optical measurements were conducted under ambient conditions. Current–voltage-luminance characteristics were recorded using a Keithley 2400 source meter and a Minolta LS160 luminance meter. Emitted light power was measured with a Newport 1936C power meter. Electroluminescence (EL) spectra were collected using a Quanta Master 40 spectrofluorometer (PTI) as a function of applied voltage. CIE coordinates were derived from the measured EL spectra using OSRAM SYLVANIA LED ColorCalculator software at the device turn-on voltage.

## Results and Discussion

### Design and Synthesis

The compounds were designed using a modular strategy inspired by previous reports on BTD-based molecules exhibiting either AIEE or TADF. Aryl, aryloxy and IDB units, previously studied in our group, are known to enhance solid-state emission by restricting molecular motion.^20,22–24^ In addition, 9,9-dimethylfluorene (FL) had been employed in symmetric BTD derivatives that display hot-exciton TADF with high solution quantum yields.^21^ Building on these precedents, we synthesized four new asymmetric 2,7-disubstituted benzothiadiazole (BTD) derivatives in which FL serves as a first donor group, while a second donor group, selected for its reported ability to promote either AIEE or TADF, was introduced at the complementary BTD position. Accordingly, FL-BTD-Ar and FL-BTD-OAr incorporate aryl and aryloxy substituents, whereas FL-BTD-IDB and FL-BTD-PXZ feature *N*-arylated donors iminodibenzyl (IDB) and phenoxazine (PXZ).

Two distinct synthetic routes were employed for their preparation (Scheme 1). For the arylated derivatives FL-BTD-Ar and FL-BTD-OAr, the substituent is first introduced at the 4-position of the BTD core. FL-BTD-Ar is obtained via Suzuki–Miyaura coupling, affording intermediate 2 in 61% yield, while FL-BTD-OAr is synthesized through nucleophilic aromatic substitution of 4-methoxyphenol, giving intermediate 3 in 54% yield. Both intermediates are subsequently subjected to a second Suzuki–Miyaura reaction to install the dimethyl-fluorenyl unit at the 7-position, affording the final products in 62% and 95% yield, respectively. In contrast, the synthesis of FL-BTD-IDB and FL-BTD-PXZ begins with the introduction of the fluorenyl donor onto the BTD core via Suzuki–Miyaura coupling. The resulting monobrominated intermediate 4 is then functionalized at the 7-position through Pd-catalyzed C–N coupling with IDB or PXZ donors, affording the target compounds in 39% and 79% yields, respectively.

**Scheme 1:**
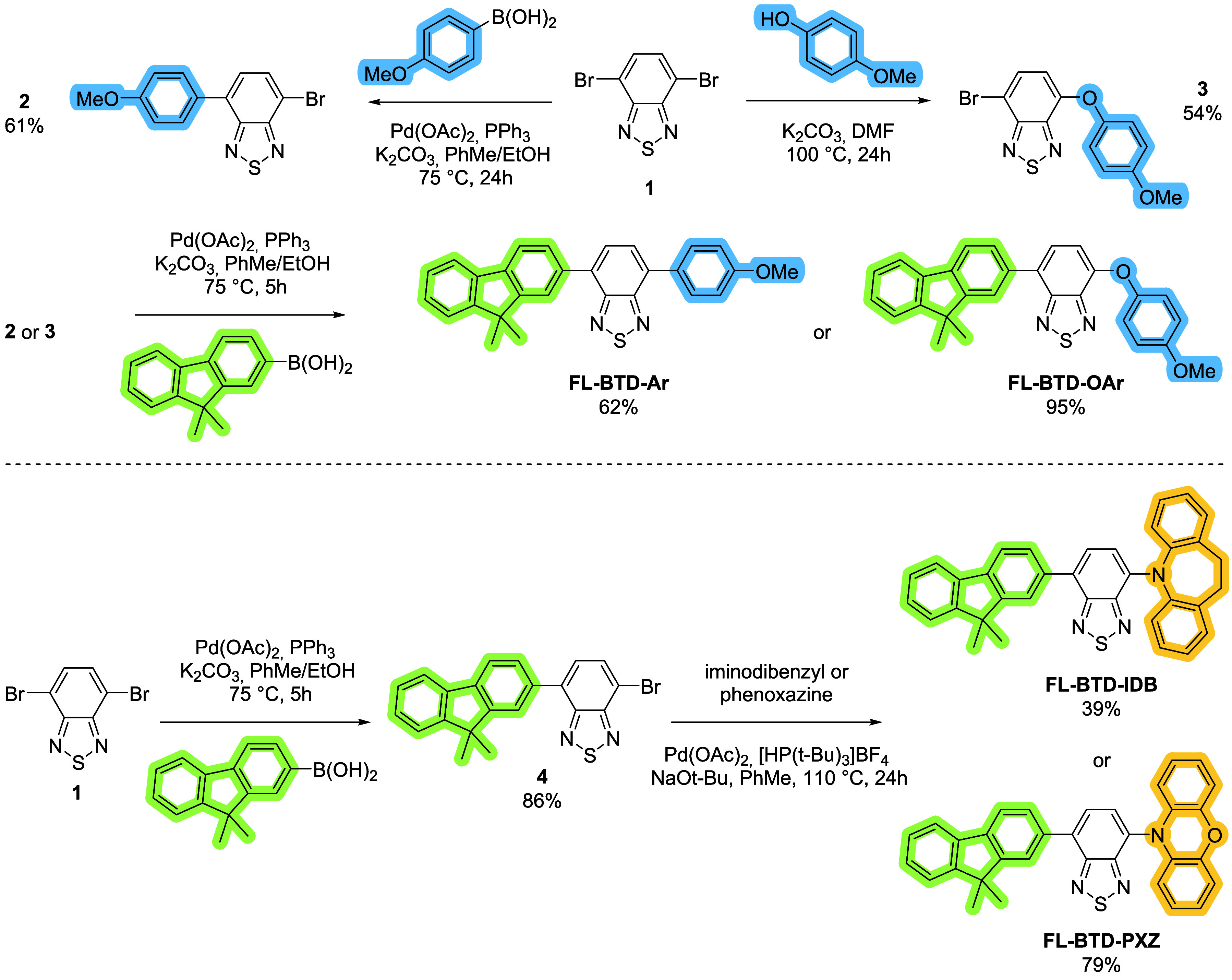
Synthesis of FL-BTD Derivatives

### Theoretical Calculations

Density functional theory (DFT) calculations under B3YLP/6-31G** level were used to estimate the HOMO and LUMO energy levels and to visualize their spatial distributions. Time-dependent DFT (TD–DFT) was further applied to compute singlet (S_1_) and triplet (T_1_) excited-state energies, the singlet–triplet energy gap (Δ*E*_ST_), and the corresponding natural transition orbitals (NTOs). These results provide insight into the excited-state character and the potential for reverse intersystem crossing (RISC), a key requirement for delayed fluorescence (Figure 1 and Table 1).

**Figure 1:**
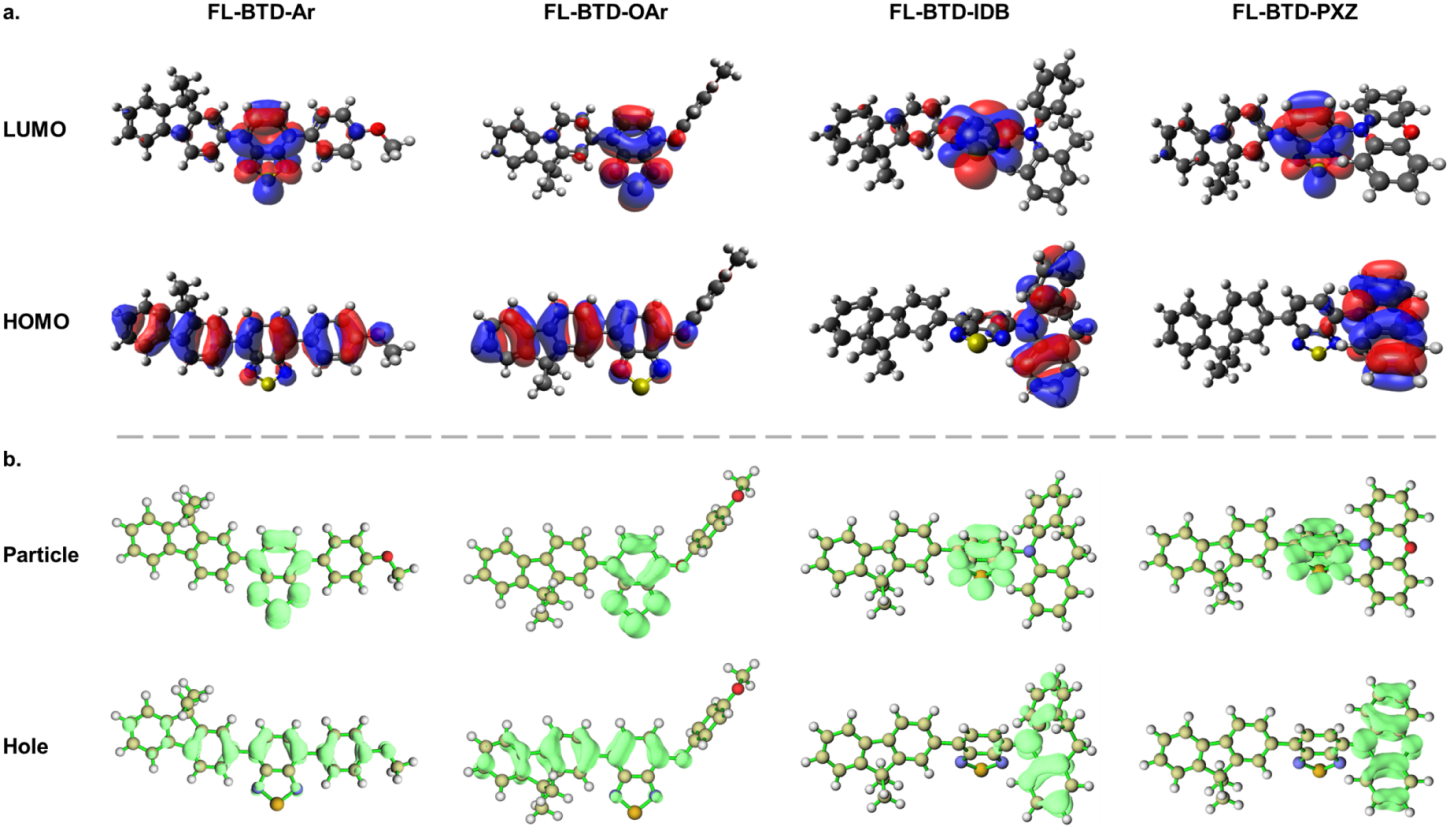
(a) HOMO and LUMO distributions of FL-BTD derivatives. (b) Natural transition orbitals (NTOs) calculated for the FL-BTD derivatives, highlighting the dominant hole and particle contributions associated with the S_0_ → S_1_ transition. The plots illustrate the spatial distribution of the frontier excitations and emphasize the extent of charge-transfer character across the series.

**Table 1:** Theoretical Simulation Data in eV for FL-BTD Derivatives

Compound	HOMO	LUMO	*E*_gap_	*E*_S_	*E*_T_	Δ*E*_ST_
FL-BTD-Ar	–5.20	–2.20	3.00	2.65	1.92	0.73
FL-BTD-OAr	–5.23	–2.07	3.16	2.77	2.02	0.75
FL-BTD-IDB	–5.01	–2.41	2.60	1.94	1.89	0.05
FL-BTD-PXZ	–4.59	–2.51	2.08	1.45	1.44	0.01

The HOMO energy levels range from −5.23 to −4.49 eV, while LUMO energies lie between −2.51 and −2.07 eV, corresponding to energy gaps (*E*_gap_) ranging from 2.08 to 3.16 eV. As expected, the incorporation of PXZ or IDB donor units raised the HOMO energy relative to the aryl or aryloxy analogues, thereby narrowing the bandgap. This trend reflects the stronger electron-donating ability of nitrogen-containing donors. In terms of orbital distribution, the LUMO of all FL-BTD derivatives is primarily localized on the electron-accepting BTD core. For FL-BTD-Ar, the HOMO is delocalized across both donor and acceptor units, whereas in FL-BTD-OAr, the oxygen atom at the D–A linkage confines the HOMO to the FL-BTD fragment with negligible contribution from the aryloxy group. In both cases, significant HOMO density on the acceptor core suggests weak charge-transfer (CT) character and a low probability of TADF emission. In contrast, FL-BTD-IDB and FL-BTD-PXZ exhibit clear spatial separation between frontier orbitals: the HOMO is confined to the donor (IDB or PXZ), while the LUMO remains localized on the BTD acceptor. This distribution indicates strong CT character, favorable for TADF.

TD-DFT results reinforce this analysis. The calculated Δ*E*_ST_ values for FL-BTD-IDB and FL-BTD-PXZ are 0.05 and 0.01 eV, respectively, sufficiently small to promote efficient RISC. NTO analysis further confirms the CT nature, with hole NTOs localized on the donor units (IDB or PXZ) and particle NTOs on the BTD core (Figure 1b). If on the one hand the presence of electrons and holes in completely separated regions of the emitter and a strong charge-transfer (CT) character are important for reducing Δ*E*_ST_ and enabling efficient RISC, on the other hand a purely CT state can significantly decrease the photoluminescence quantum yield, compromising the emitter’s performance in optoelectronic devices.^35–37^ By contrast, FL-BTD-Ar and FL-BTD-OAr display much larger Δ*E*_ST_ values (>0.7 eV) and predominantly local excitation (LE) character in their NTOs. This suggests their emission is dominated by prompt fluorescence, with minimal or absent contribution from triplet harvesting pathways.

### Photophysical and Electrochemical Properties

The photophysical properties of the FL-BTD derivatives were examined in various solvents (toluene, THF, chloroform, acetonitrile, and ethanol). Absorption and emission spectra are presented in Figure 2, with the corresponding data summarized in Table 2. The compounds exhibit absorption maxima between 397 and 505 nm. As expected, IDB- and PXZ-substituted derivatives display red-shifted absorption compared to the aryl- and aryloxy-substituted analogues, consistent with the smaller bandgaps predicted by DFT. FL-BTD-Ar, FL-BTD-OAr, and FL-BTD-IDB exhibited relatively high molar extinction coefficients on the order of 10^4^ L mol^–1^ cm^–1^, whereas FL-BTD-PXZ shows weaker absorption, with coefficients in the 10^3^ L mol^–1^ cm^–1^ range, all associated with π–π* transitions. Notably, FL-BTD-PXZ demonstrates distinct solvatochromic behavior compared to the other derivatives. While FL-BTD-Ar, FL-BTD-OAr, and FL-BTD-IDB show only minor absorption shifts (Δλ_abs_ = 3–9 nm), indicative of negligible ground-state charge transfer, FL-BTD-PXZ exhibits a pronounced 33 nm shift across solvents. This behavior suggests a higher degree of ground-state charge separation for the PXZ-substituted derivative. Optical bandgaps estimated from the absorption onsets fall within 2.06 to 2.68 eV, values that are suitable for application as emitters in optoelectronic devices.

**Figure 2:**
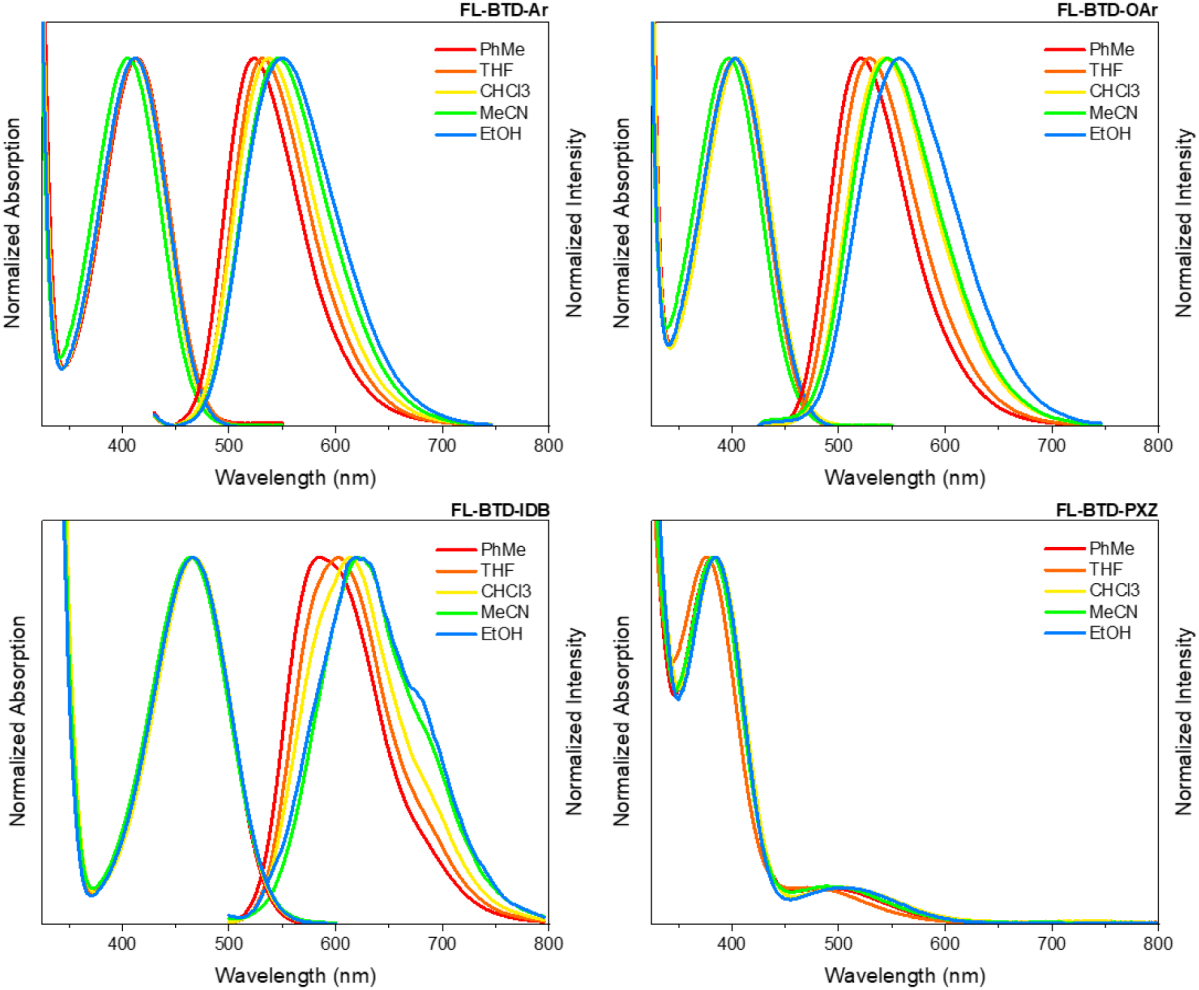
Normalized UV–vis absorption and emission spectra of FL-BTD derivatives in different organic solvents (PhMe: toluene, THF: tetrahydrofuran, CHCl_3_: chloroform, MeCN: acetonitrile, and EtOH: ethanol).

**Table 2:** Photophysical Data for FL-BTD Derivatives in Solution^a^

Compound		FL-BTD-Ar	FL-BTD-OAr	FL-BTD-IDB	FL-BTD-PXZ
λ_abs_ (nm)	Toluene	414	404	465	505
	THF	413	402	465	490
	Chloroform	411	405	467	505
	Acetonitrile	405	397	464	472
	Ethanol	411	403	466	490
Δλ_abs_ (nm)		9	7	3	33
ε_THF_ (cm^–1^ mol^–1^ L)		23,564	9653	11,457	1400
*E*_gap_ (eV) THF		2.63	2.68	2.32	2.06
λ_em_ (nm)	Toluene	522	519	585	–
	THF	529	528	603	–
	Chloroform	535	543	614	–
	Acetonitrile	545	548	621	–
	Ethanol	550	555	620	–
Δλ_em_ (nm)		28	36	36	–
Δλ_St_ (cm^–1^)	Toluene	4998	5485	4411	–
	THF	5309	5936	4922	–
	Chloroform	5639	6275	5127	–
	Acetonitrile	6343	6941	5449	–
	Ethanol	6149	6796	5330	–
ϕ_PL_	Toluene	0.65	0.42	0.10	–
	THF	0.67	0.65	0.08	–

a λ_abs_ and λ_em_ are the absorption and emission maxima, Δλ_abs_ and Δλ_em_ are the solvatochromism in the ground and excited states, respectively, ε is the molar extinction coefficient, *E*_gap_ is the optical band gap, Δλ_ST_ is Stokes shift, and ϕ_PL_ is the PLQY.

FL-BTD-Ar, FL-BTD-OAr, and FL-BTD-IDB exhibit fluorescence in solution, while FL-BTD-PXZ shows no significant emission in any tested solvents. The aryl- and aryloxy-substituted derivatives emit in the green to yellow region (519–555 nm), whereas the IDB derivative presented fluorescence in the yellow to orange region (585–620 nm). For the three emissive compounds, large Stokes shifts and pronounced positive solvatochromism are observed, consistent with a stabilized excited state of intramolecular charge-transfer (ICT) character, as expected for this class of compounds.^38^ Photoluminescence quantum yields (PQLYs) determined in toluene and THF range from 0.08 to 0.67, following the order: FL-BTD-Ar > FL-BTD-OAr > FL-BTD-IDB > FL-BTD-PXZ (nonemissive). Based on the NTOs shown in Figure 1b, the phenoxazine-substituted compound exhibits electrons and holes localized in highly separated regions, resulting in an excited state with very strong charge-transfer (CT) character. Although this configuration is favorable for reducing Δ*E*_ST_ and could in principle promote efficient reverse intersystem crossing, the CT character appears to be overly dominant. When the excited state becomes too pure CT, its radiative decay rate decreases dramatically, and nonradiative decay pathways prevail.^35–37^ As a result, the photoluminescence quantum yield is severely suppressed, which explains why no measurable PL emission was observed for this compound. It is important to note that the absence of photoluminescence has also been reported in other symmetric and asymmetric compounds that combine phenoxazine, as donor, with benzothiadiazole, as acceptor.^20^

To evaluate AIEE behavior, fluorescence measurements of the FL-BTD derivatives were conducted in THF/water mixtures with increasing water fraction (*f*_w_) (Figure 3). For FL-BTD-Ar, a progressive decrease in ϕ_PL_ from 0.65 to 0.18 was observed as *f*_w_ increased, indicating the absence of AIEE. Nonetheless, emission was not completely quenched in highly aqueous mixtures, suggesting partial resistance to aggregation-induced quenching. In the case of FL-BTD-OAr, an initial decrease in fluorescence intensity was observed as the water fraction increased, attributed to solvent polarity effects on the charged excited state. However, at *f*_w_ = 80%, a sharp increase in emission occurred. ϕ_PL_ values dropped from 0.41 in pure THF to 0.03 at *f*_w_ = 70%, followed by recovery to 0.30 at *f*_w_ = 99%, representing a 10-fold enhancement upon aggregation and recovery of ∼70% of the initial emission. Similarly, FL-BTD-IDB displayed emission recovery upon aggregation. The ϕ_PL_ decreased from 0.08 at *f*_w_ = 0% to 0.01 at *f*_w_ = 70% and fully recovered to 0.08 at *f*_w_ = 85%, confirming its AIEE character. In contrast, FL-BTD-PXZ remained nonemissive under all conditions, consistent with its behavior in solution. These results highlight the influence of the substitution pattern on the emission upon aggregation, where aryloxy and IDB groups promote AIEE in BTD derivatives, whereas the simple 4-methoxyaryl group in FL-BTD-Ar leads to progressive fluorescence quenching. Dynamic light scattering (DLS) measurements at *f*_w_ = 80% for the AIEE-active compounds revealed particle diameters of 311 nm (FL-BTD-OAr) and 247 nm (FL-BTD-IDB) (Figure S2). These findings confirm that emission recovery is directly correlated with aggregate formation in THF/water mixtures.

**Figure 3:**
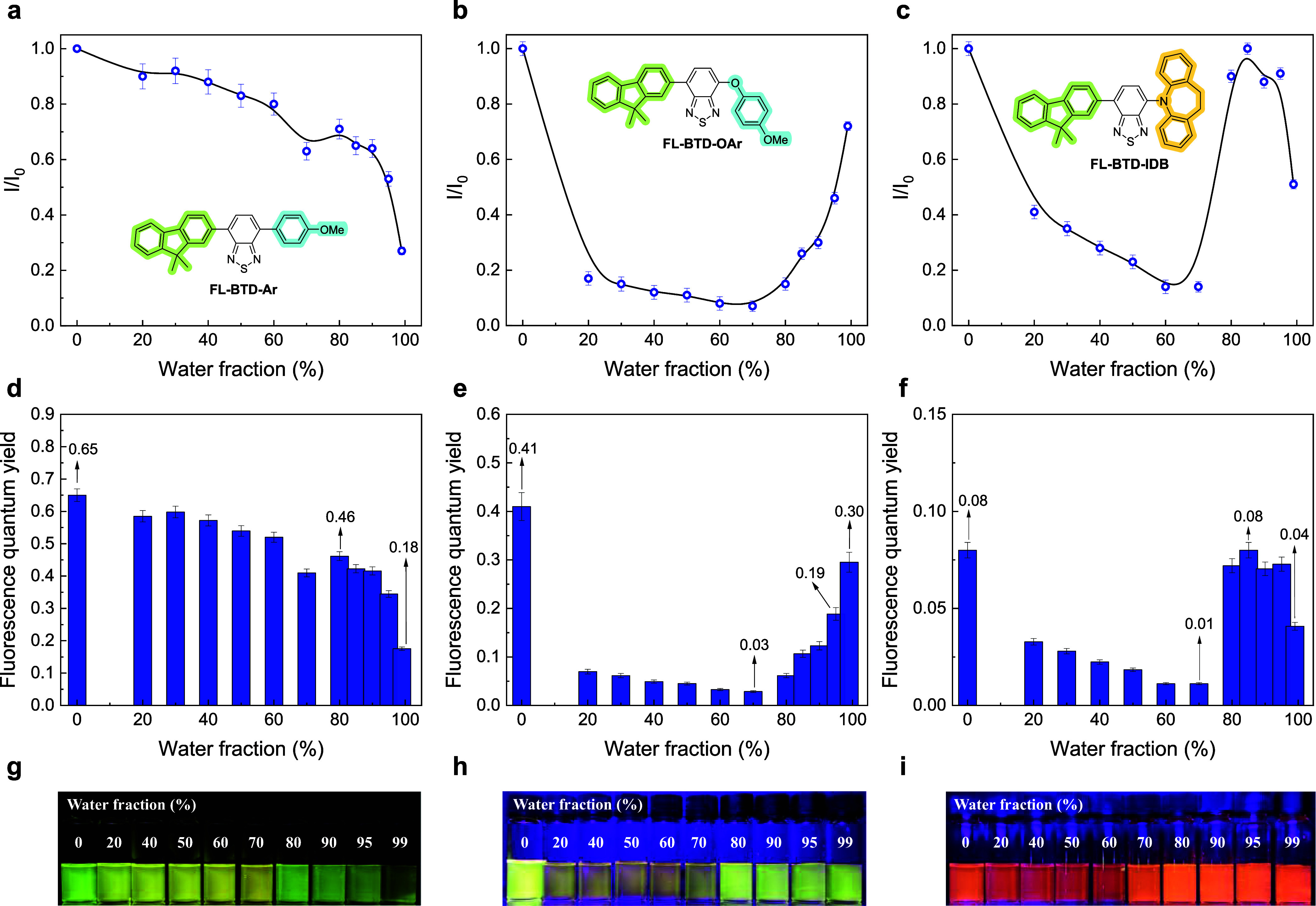
Relative fluorescence intensity (a–c), PQLY (d–f), and pictures (g–i) for FL-BTD derivatives in THF/water mixtures with different *f*_w_.

Additional insight into the AIEE mechanism was obtained from single-crystal X-ray diffraction analyses of FL-BTD-OAr and FL-BTD-Ar (Figures 4 and S3, Table S1–S5). FL-BTD-OAr crystallizes with two independent molecules in the asymmetric unit, adopting nearly perpendicular arrangements between the BTD planes (86.6°). The donor–acceptor dihedral angles are 15.5° (FL|BTD) and 80.6° (BTD|OAr), resulting highly twisted conformations. These geometries generate intermolecular distances of 5.3–8.6 Å, effectively preventing π-π stacking and favoring radiative decay. Additionally, short contacts such as N···S (3.20 Å and 3.25 Å) and C···S (3.41 Å and 3.44 Å) help lock the molecular conformation and restrict intramolecular motions. By contrast, the AIEE-inactive FL-BTD-Ar adopts a less twisted geometry, with donor–acceptor dihedral angles of 41.8° and 49.0°. This conformation facilitates closer packing and enables π–π interactions between BTD units, with centroid–centroid distances of 3.85 Å. Such packing promotes nonradiative decay pathways, explaining the pronounced fluorescence quenching observed at high water fractions for this compound.

**Figure 4:**
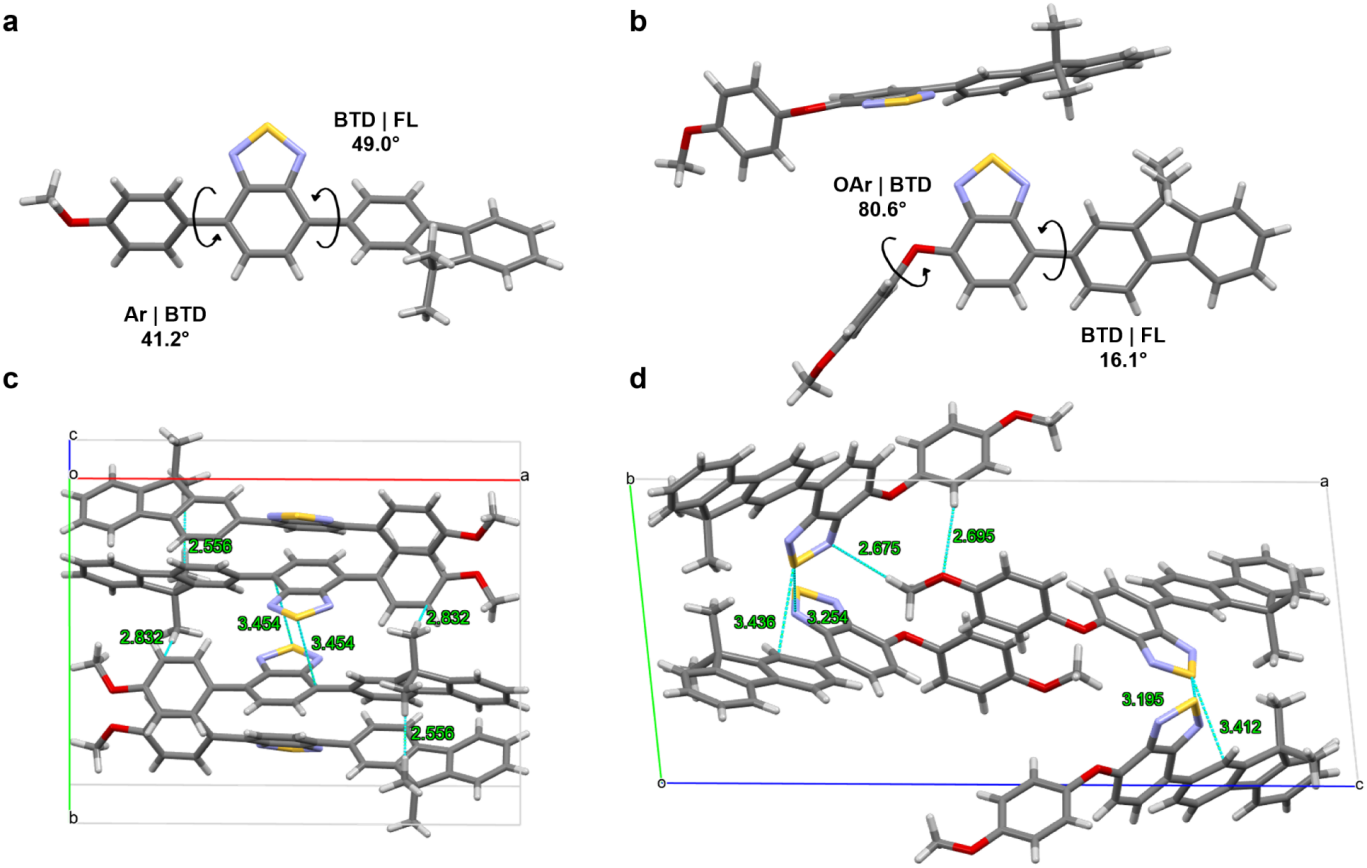
Single-crystal structures of FL-BTD-Ar (a) and FL-BTD-OAr (b), showing dihedral angles between FL, BTD, and Ar/OAr units, and their respective packing arrangements (c,d), and short contact distances.

Electrochemical properties of the FL-BTD derivatives were investigated by cyclic voltammetry to estimate their HOMO and LUMO energy levels. The voltammograms are shown in Figures S4 and S5, and the estimated values are summarized in Table 3. The HOMO energies range from −5.77 to −6.01 eV, while the LUMO energies lie between −2.87 and to −3.38 eV. The narrowest gap (1.96 eV) is observed for FL-BTD-PXZ, consistent with its strong electron-donating character and red-shifted absorption. Overall, the electrochemical results show good agreement with theoretical predictions and optical bandgaps derived from absorption onsets, supporting the rational design strategy based on donor strength modulation.

**Table 3:** Photophysics in Solid State and Electrochemical Data for FL-BTD Derivatives

Compound	λ_abs_ (nm)^a^	λ_em_ (nm)^a^	ϕ_PL_ ^a^	HOMO/LUMO (eV)^b^	*E*_opt_ (eV)^c^	τ_p_ (ns)^d^	τ_d_ (μs)^d^
FL-BTD-Ar	425	517	0.60	–5.84/–3.33	2.40	7.9	–
FL-BTD-OAr	433/484	533	0.70	–5.85/–3.38	2.33	13.5	–
FL-BTD-IDB	444/532	602	0.30	–5.77/–2.87	2.06	11.0	1.03
FL-BTD-PXZ	567	632	0.03	–6.01/–3.16	1.96	–	–

a Measured in powder.

b Calculated from the empirical formula HOMO = −(*E*_ox_ – Δ*E*_ref_ + 4.4) eV, LUMO = −(*E*_ref_ – Δ*E*_ref_ + 4.4) eV, Δ*E*_ref_ = 0.07 eV.

c Estimated from the onset of the absorption edge of the powders.

d PL lifetimes of prompt and delayed decay components in films at room temperature.

The solid-state photophysical properties of the FL-BTD derivatives are summarized in Table 3 and illustrated in Figure 5. Diffuse reflectance UV–vis spectra show broad absorption spanning the UV–visible region. The optical bandgaps were determined as 2.40 eV (FL-BTD-Ar), 2.42 eV (FL-BTD-OAr), and 2.06 eV (FL-BTD-IDB). Emission maxima are compared to those observed in solution, ranging from green to orange: FL-BTD-Ar (517 nm), FL-BTD-OAr (533 nm), FL-BTD-IDB (602 nm), and FL-BTD-PXZ (632 nm). Notably, FL-BTD-PXZ, which was nonemissive in solution, exhibited very weak but detectable fluorescence in the solid state (ϕ_PL_ = 0.03), suggesting aggregation induced emission (AIE), although less efficient than the other derivatives. Solid-state ϕ_PL_ values follow the order FL-BTD-OAr (0.70) > FL-BTD-Ar (0.60) > FL-BTD-IDB (0.30). Compared to their efficiencies in toluene (0.42, 0.65, and 0.10, respectively), FL-BTD-OAr and FL-BTD-IDB exhibited significant enhancements upon aggregation, consistent with AIEE behavior, while FL-BTD-Ar shows a slight decrease, indicating weaker aggregation-induced effects.

**Figure 5:**
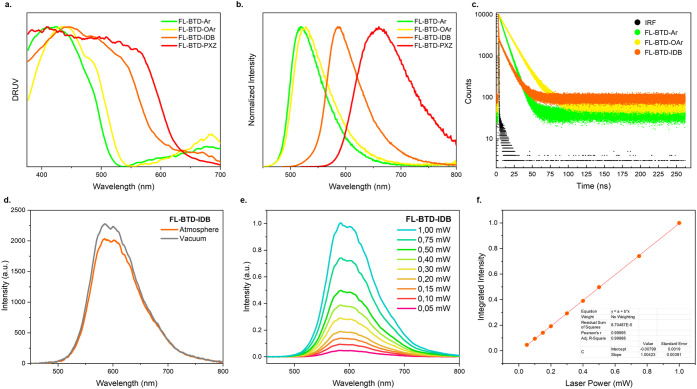
Solid–state spectra of FL-BTD derivatives. (a) Normalized DRUV, (b) steady–state fluorescence emission, (c) PL spectrum with atmospheric air and under vacuum for FL-BTD-IDB film, and (d) transient PL decay curves.

To gain further insight into the emission mechanisms, particularly the involvement of triplet states, additional measurements were carried out under vacuum and in the presence of O_2_, as well as excitation-power-dependent studies (Figures 5c, S6 and S7) and time-resolved PL measurements (Figure 5d). The emission of FL-BTD-Ar and FL-BTD-OAr remained unchanged under vacuum and O_2_ conditions, confirming their emission arises exclusively from singlet states. In contrast, FL-BTD-IDB displayed stronger emission under vacuum than in the presence of oxygen, indicating that it can harvest triplet excitons and exhibits triplet-based luminescence. FL-BTD-Ar and FL-BTD-OAr exhibited monoexponential decays with prompt fluorescence lifetimes of 7.9 and 13.5 ns, respectively, and no detectable delayed components, confirming that their emission arises exclusively from prompt fluorescence. In contrast, FL-BTD-IDB displayed a biexponential decay, with lifetimes of 11 ns and 1.03 μs, revealing the presence of a delayed fluorescence component. Excitation-power-dependent experiments (Figure 5e and f) further showed a linear relationship with a slope ≈1 between excitation power and emission intensity, ruling out triplet–triplet annihilation (TTA) as the origin of the long-lived emission.^39^ Collectively, these findings confirm the occurrence of TADF in FL-BTD-IDB. Thus, the molecular design of FL-BTD-IDB can be considered successful, as the combined incorporation of fluorenyl and IDB units imparts both AIEE and TADF characteristics to the BTD scaffold.

### Electroluminescent Properties

Multilayer OLEDs were fabricated using FL-BTD-Ar, FL-BTD-OAr (AIEE-active), and FL-BTD-IDB (combining AIEE and TADF) as emissive layers. Owing to the distinct photophysical properties of these derivatives, different device architectures were employed. For FL-BTD-Ar and FL-BTD-OAr, the best results were obtained with the following configuration: ITO (150 nm)/MoO_3_ (5 nm)/β-NPB (30 nm)/BCPO:emitter (30 wt %, 20 nm)/Bphen (40 nm)/LiF (0.5 nm)/Al (70 nm). For FL-BTD-IDB, the configuration was ITO (150 nm)/MoO_3_ (5 nm)/β-NPB (68 nm)/Bepq_2_:emitter (30 wt %, 20 nm)/LiF (0.5 nm)/Al (70 nm). BCPO and Bepq_2_ were chosen as host materials to ensure efficient exciton confinement and energy transfer to the FL-BTD derivatives in a host–guest system.^40^ The electroluminescence (EL) spectra recorded at turn-on voltages, along with the brightness and power density curves, are shown in Figure 6, with the corresponding device parameters summarized in Table 4.

**Figure 6:**
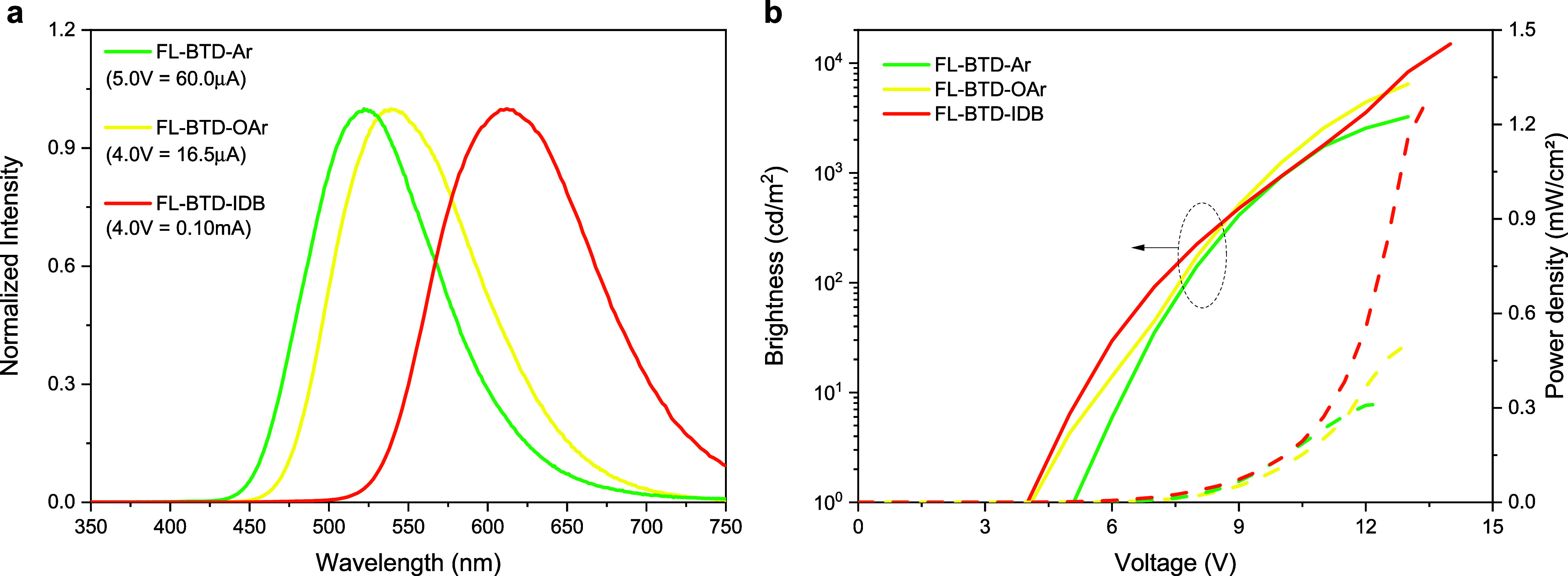
Electroluminescence performance of FL-BTD derivative-based OLEDs. (a) EL spectra recorded at respective turn-on voltages. (b) Characteristic curves showing brightness versus applied voltage and emitted light power versus applied voltage.

**Table 4:** Summarized OLED Characteristics

Compound	λ_em_ (nm)	*V*_on_ (V)	Brightness (cd m^–2^)^a^	Power density (mW cm^–2^)^a^	EQE (%)^b^	CIE (*x*, *y*)
FL-BTD-Ar	524	5.0	3250	0.31	0.3	0.29, 0.52
FL-BTD-OAr	538	4.0	6400	0.50	0.4	0.36, 0.57
FL-BTD-IDB	610	4.0	15,000	1.28	0.7	0.57, 0.43

a Maximum values.

b Measured at 1 kcd m^–2^.

The OLEDs exhibited emission maxima at 524 nm (FL-BTD-Ar), 538 nm (FL-BTD-OAr), and 610 nm (FL-BTD-IDB), corresponding to green emission for the first two derivatives and orange emission for the latter, with CIE coordinates of (0.29, 0.52), (0.36, 0.57), and (0.57, 0.43), respectively (Figure S8). Turn-on voltages of 4.0 V were observed for FL-BTD-OAr and FL-BTD-IDB, while FL-BTD-Ar required 5.0 V. Maximum brightness reached 3250 cd m^–2^ (FL-BTD-Ar), 6400 cd m^–2^ (FL-BTD-OAr), and 15,000 cd m^–2^ (FL-BTD-IDB). External Quantum Efficiencies (EQEs), calculated from the light power emission versus applied voltage curves,^41^ were 0.3% (FL-BTD-Ar), 0.4% (FL-BTD-OAr), and 0.7% (FL-BTD-IDB). Although the EQE values obtained in our devices are modest, this is expected given that no optimization of the OLED architecture was carried out. In general, improvements could be achieved through the selection of more suitable host materials, adjustments in layer thicknesses, or refinements in charge-injection and carrier-balance conditions. It should be emphasized, however, that device optimization was beyond the scope of this work; our focus was to correlate the electroluminescence behavior with the photophysical properties measured in solution and neat films. Importantly, devices performance trends directly reflect the photophysical properties of the emitters. FL-BTD-Ar suffers from partial ACQ, limiting its electroluminescence efficiency. FL-BTD-OAr, in contrast, benefits from AIEE, which enhances brightness and EQE. The best-performing device, based on FL-BTD-IDB, combines AIEE and TADF, where the former enhances solid-state emission, while the latter enables triplet harvesting via RISC. This dual mechanism accounts for the highest luminance and EQE among the series. Although the efficiencies remain modest compared with the symmetric “hot-exciton” FL-BTD-FL,^21^ these results underscore the critical role of molecular design in tuning solid-state emission and suggest that further optimization of device architecture and doping strategies could substantially improve performance.

## Conclusion

In summary, we have developed and systematically investigated four novel asymmetric fluorenyl–benzothiadiazole derivatives aimed at achieving efficient solid-state emission through the strategic integration of strong electron donors with a robust acceptor core. By combining experimental and computational studies, we established clear structure–property relationships with a particular focus on AIEE and TADF. Twisted donor groups such as aryloxy and IDB effectively promoted AIEE, while FL-BTD-IDB uniquely combined both AIEE and TADF, exhibiting a delayed fluorescence component and a small singlet–triplet energy gap. In contrast, FL-BTD-Ar showed moderate but nonenhanced emission under aggregation, and FL-BTD-PXZ, although nonemissive in solution, displayed weak solid-state emission, indicative of limited AIE. These findings emphasize the potential of asymmetric donor–acceptor molecular design to overcome ACQ and enable multifunctional emitters capable of triplet harvesting. The electroluminescent results of the corresponding OLEDs closely mirrored the solid-state photophysics: FL-BTD-Ar, affected by partial aggregation quenching, displayed the lowest performance; FL-BTD-OAr benefited from AIEE, achieving improved brightness and efficiency; and the best-performing device, based on FL-BTD-IDB, combined AIEE and TADF to deliver in the highest brightness (15,000 cd m^–2^) and EQE (0.7%). Although efficiencies remain modest compared to BTD-based TADF OLEDs, the results clearly demonstrate that even in nonoptimized device architectures, the synergistic interplay of AIEE and TADF can be directly translated into electroluminescent performance. This work highlights asymmetric donor–acceptor engineering as a promising design strategy for multifunctional emitters and provides a foundation for future optimization of device architectures and doping strategies.

## Supplementary Material



## Data Availability

The data underlying this study are available in the published article and its Supporting Information.

## Notes

Natural transition orbital (NTO) analysis of the excited states, hydrodynamic diameters in aqueous aggregates determined by DLS, ORTEP crystal structures, electrochemical measurements, additional photophysical characterization, CIE coordinates and energy-level diagrams of OLED devices, and spectroscopic data (^1^H, ^13^C (APT) NMR, and FTIR spectra) of all synthesized compounds (PDF)
