# Supplementary material for: Interplay of Aggregation-Induced Enhanced Emission and Thermally Activated Delayed Fluorescence in Asymmetric Fluorenyl–Benzothiadiazole Derivatives
Source: ACS Phys Chem Au. 2025 Dec 15;6(1):215–26. doi: 10.1021/acsphyschemau.5c00118 (PMC12856649; doi:10.1021/acsphyschemau.5c00118)
Supplement: Supplementary file 1 [file pg5c00118_si_001.pdf]

Supporting Information

Interplay of aggregation-induced enhanced emission and thermally activated delayed fluorescence in asymmetric fluorenyl–benzothiadiazole derivatives

Interplay of aggregation-induced enhanced emission and thermally activated delayed fluorescence in asymmetric fluorenyl–benzothiadiazole derivatives

Carolina Vesga-Hernández [a], *Rafael S. Carvalho* [b], *Aline M. Santos* [a,b], *Marlin J. P. Peñafiel* [a], *Luiz Maqueira* [a], *Davi F. Back* [c], *Ricardo Q. Aucélio* [a], *Fabiano Rodembusch* [d], *Flavio Franchello* [e], *Edson Laureto* [e], *Marco Cremona* [b], *and Jones Limberger* [a*]

*E-mail: limberger@puc-rio.br

[a]Department of Chemistry, Pontifícia Universidade Católica do Rio de Janeiro, 22451–900, Rio de Janeiro, RJ, Brazil.

[b]Department of Physics, Pontifícia Universidade Católica do Rio de Janeiro, 22451–900, RJ, Brazil.

[c]Department of Chemistry, Universidade Federal de Santa Maria, 97105–900, Santa Maria, RS, Brazil.

[d]Institute of Chemistry, Universidade Federal do Rio Grande do Sul, 91501–970, Porto Alegre, RS, Brazil.

[e]Department of Physics, State University of Londrina, 86057–970, Londrina, Paraná, Brazil.

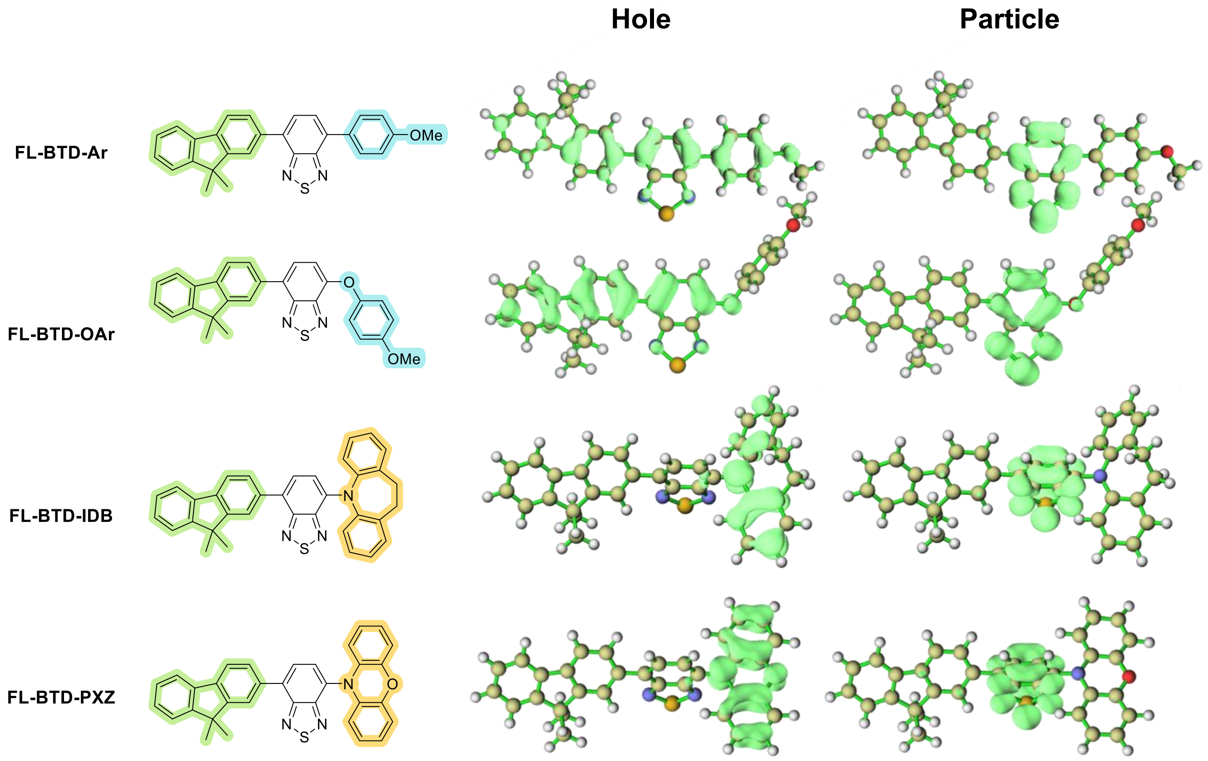

Figure S 1. Natural transition orbital (NTO) pairs for the excited states.

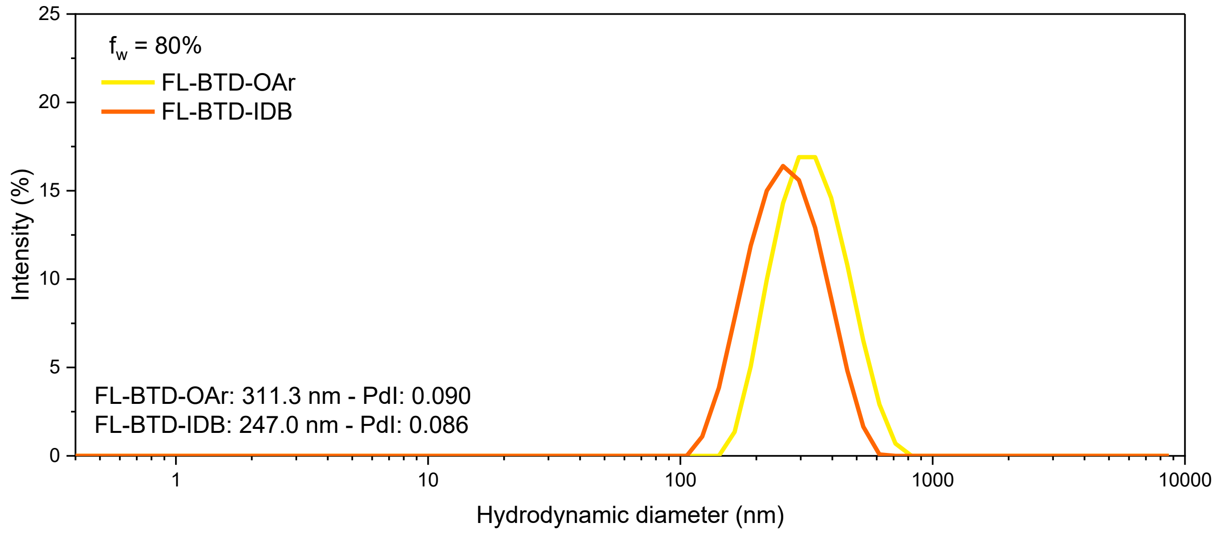

Figure S 2. Hydrodynamic diameters of FL-BTD derivatives as aqueous aggregates determined by DLS.

Table S 1. Crystal data, data collection and refinements of molecules FL-BTD-Ar and FL-BTD-OAr

	FL-BTD-Ar	FL-BTD-OAr
**Empirical formula**	$C_{28}H_{22}N_2OS$	$C_{56}H_{44}N_4O_4S_4$ *
**Formula weight**	434.54	901.07
**Crystal system, space group**	Monoclinic, *P* 21/c	Triclinic, *P*-1
**T / K**	100(2)K	296(2)K
**Radiation, λ / Å**	0.71073	1.54178
**Unit cell (Å) dimensions *a*,**	18.7392(6)	8.0515(6)
***b*,**	14.7782(4)	11.1009(8)
c	7.7873(2)	25.2918(18)
**α, (°)**	90	96.125(3)
**β**	91.0780(10)	97.954(4)
**γ**	90	90.598(4)
***V* (Å^3)**	2156.17(11)	2225.3(3)
***Z*, Calculated density (g.cm^{-3})**	4, 1.339	2, 1.345
**Absorption coefficient (mm^{-1})**	0.174	1.520
***F* (000)**	912	944
**Crystal size (mm)**	0.30 x 0.21 x 0.20	0.109 x 0.03 x 0.02
**Theta range for data collection**	2.17 – 30.57	4.01 – 83.13
**Index ranges**	$-26 \leq h \leq 25$	$-10 \leq h \leq 9$
	$-21 \leq k \leq 21$	$-14 \leq k \leq 13$
	$-11 \leq l \leq 10$	$-32 \leq l \leq 31$
**Reflections collected / unique**	34201 / 6589 [R(int) = 0.0254]	20041 / 9925 [R(int) = 0.0647]
**Completeness to theta max**	99.6%	99.6 %
**Absorption correction**	Multi-scan	Multi-scan
**Max. and min. transmission**	0.9660 and 0.9396	0.9861 and 0.9629

**Refinement method**	Full-matrix least-squares on F^2	Full-matrix least-squares on F^2
**Data / restraints / parameters**	6589/ 0 / 289	9925 / 0 / 596
**Goodness-of-fit on F^2**	1.032	2.108
**Final R indices [I > 2σ(I)]**	R1 = 0.0368, wR2 = 0.0983	R1 = 0.1771, wR2 = 0.1106
**R indices**	R1 = 0.0429, wR2 = 0.1021	R1 = 0.2103, wR2 = 0.2310
**Largest diff. peak and hole (e.Å^{-3})**	0.456 and -0.297	1.097 and -0.791

* Two independent units per unit cell

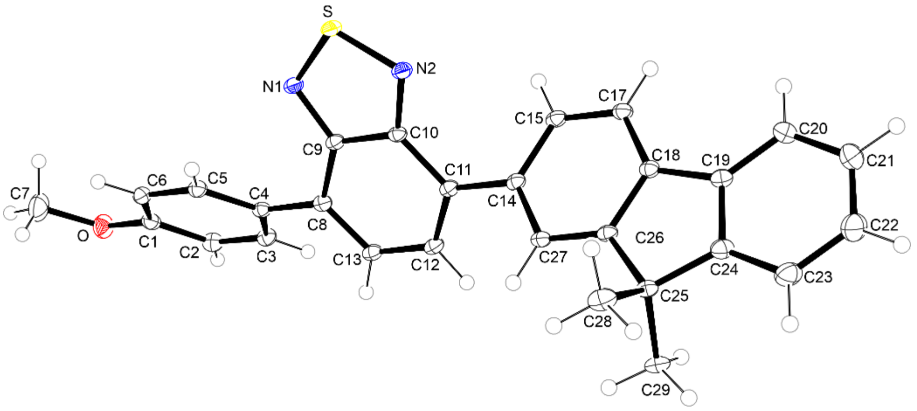

FL-BTD-Ar

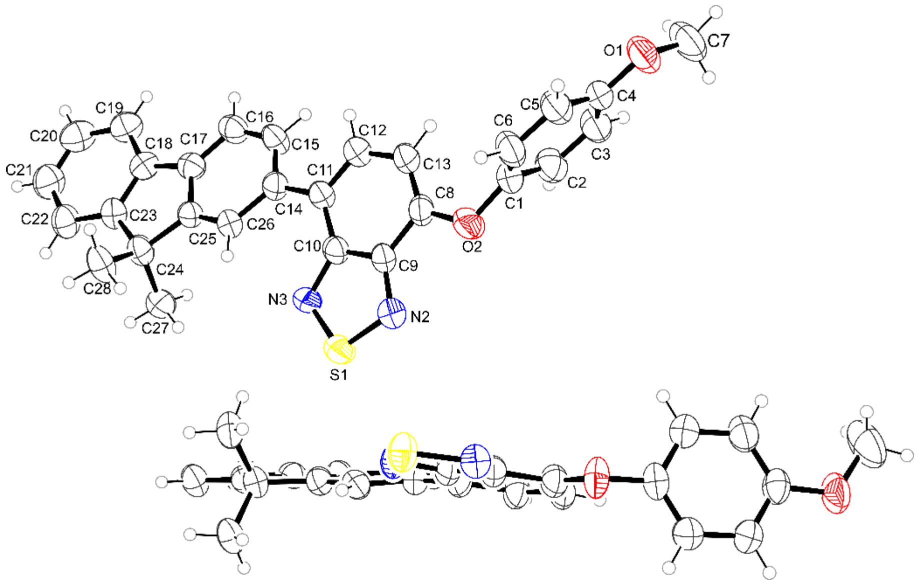

FL-BTD-OAr

Figure S 3. ORTEP diagrams for FL-BTD-Ar and FL-BTD-OAr with ellipsoids drawn at 50% probability.

Table S 2. Short contacts [Å] in the crystal structures of FL-BTD-Ar.

Atom1	Atom2	Length	Length-VdW	Symm. op. 1	Symm. op. 2
O	H21	2.62	-0.1	x, y, z	-1+x, y, z
S	C11	3.454	-0.046	x, y, z	1-x, 1-y, 1-z
C6	H28B	2.832	-0.068	x, y, z	1-x, 1-y, 1-z
H29C	C17	2.556	-0.344	x, y, z	x, 1.5-y, -1/2+z

Table S 3. Short contacts [Å] in the crystal structures of FL-BTD-OAr

Atom1	Atom2	Length	Length-VdW	Symm. op. 1	Symm. op. 2
H13	H6A	2.394	-0.006	x, y, z	-1+x, -1+y, z
N3	S1A	3.254	-0.096	x, y, z	-1+x, y, z
C26	S1A	3.436	-0.064	x, y, z	-1+x, y, z
H6	H13A	2.384	-0.016	x, y, z	x, -1+y, z
S1	N3A	3.195	-0.155	x, y, z	x, y, z
S1	C26A	3.412	-0.088	x, y, z	x, y, z
O1	H2A	2.695	-0.025	x, y, z	2-x, 1-y, 1-z
H7A	N2A	2.675	-0.075	x, y, z	2-x, 1-y, 1-z
N2	H7A1	2.651	-0.099	x, y, z	2-x, 2-y, 1-z
H2	O1A	2.713	-0.007	x, y, z	2-x, 2-y, 1-z
C21A	H27B	2.879	-0.021	x, y, z	-1+x, y, z

Table S 4. Values of selected bond lengths for FL-BTD-Ar and FL-BTD-OAr.

FL-BTD-Ar		FL-BTD-OAr	
**Atoms**	**Bond**	**Atoms**	**Bond**
S-N(2)	1.6117(9)	S(1)-N(3)	1.591(7)
S-N(1)	1.6129(9)	S(1)-N(2)	1.618(7)
O-C(1)	1.3690(13)	S(1A)-N(3A)	1.613(7)
O-C(7)	1.4224(16)	S(1A)-N(2A)	1.617(7)
N(1)-C(9)	1.3543(12)	O(2A)-C(8A)	1.358(10)
N(2)-C(10)	1.3546(12)	O(2A)-C(1A)	1.382(10)
C(9)-C(8)	1.4331(13)	O(1A)-C(4A)	1.369(10)
C(9)-C(10)	1.4349(14)	O(1A)-C(7A)	1.414(16)

C(10)-C(11)	1.4304(13)	O(1)-C(4)	1.358(10)
C(8)-C(13)	1.3787(13)	O(1)-C(7)	1.405(13)
C(8)-C(4)	1.4745(14)	N(3)-C(10)	1.359(10)
C(18)-C(17)	1.3914(14)	N(3A)-C(10A)	1.344(10)
C(18)-C(26)	1.4066(13)	O(2)-C(8)	1.354(10)
C(18)-C(19)	1.4637(14)	O(2)-C(1)	1.418(10)
C(4)-C(5)	1.3985(14)	N(2A)-C(9A)	1.328(12)
C(4)-C(3)	1.4038(14)	C(14A)-C(26A)	1.410(11)
C(17)-C(15)	1.3856(15)	C(14A)-C(15A)	1.417(12)
C(12)-C(11)	1.3791(14)	C(14A)-C(11A)	1.471(10)
C(12)-C(13)	1.4209(14)	C(23A)-C(22A)	1.376(11)
C(26)-C(27)	1.3808(14)	C(23A)-C(18A)	1.390(12)
C(26)-C(25)	1.5251(13)	C(23A)-C(24A)	1.526(11)
C(14)-C(15)	1.4043(13)	N(2)-C(9)	1.353(12)
C(14)-C(27)	1.4080(13)	C(25)-C(26)	1.366(12)
C(14)-C(11)	1.4789(14)	C(25)-C(17)	1.407(12)
C(19)-C(20)	1.3935(15)	C(25)-C(24)	1.534(10)
C(19)-C(24)	1.4073(14)	C(26)-C(14)	1.415(11)
C(3)-C(2)	1.3828(15)	C(14)-C(15)	1.396(13)
C(6)-C(5)	1.3897(15)	C(14)-C(11)	1.479(11)
C(6)-C(1)	1.3929(15)	C(10)-C(9)	1.418(11)
C(25)-C(24)	1.5224(15)	C(10)-C(11)	1.437(12)
C(25)-C(29)	1.5319(14)	C(24A)-C(25A)	1.519(11)
C(25)-C(28)	1.5405(14)	C(24A)-C(27A)	1.526(12)
C(2)-C(1)	1.3959(15)	C(24A)-C(28A)	1.537(14)
C(24)-C(23)	1.3868(16)	C(16)-C(17)	1.368(12)
C(20)-C(21)	1.3913(17)	C(16)-C(15)	1.400(13)
C(23)-C(22)	1.3967(18)	C(11A)-C(12A)	1.377(11)
C(21)-C(22)	1.3926(19)	C(11A)-C(10A)	1.445(12)
		C(26A)-C(25A)	1.390(11)
		C(10A)-C(9A)	1.425(11)
		C(18A)-C(19A)	1.400(12)
		C(18A)-C(17A)	1.452(11)
		C(8)-C(13)	1.354(14)

C(8)-C(9)	1.437(11)
C(17A)-C(16A)	1.384(12)
C(17A)-C(25A)	1.411(12)
C(22)-C(21)	1.391(15)
C(22)-C(23)	1.394(11)
C(22A)-C(21A)	1.390(14)
C(17)-C(18)	1.455(11)
C(15A)-C(16A)	1.389(12)
C(11)-C(12)	1.370(11)
C(9A)-C(8A)	1.444(11)
C(1)-C(6)	1.358(15)
C(1)-C(2)	1.365(14)
C(18)-C(23)	1.405(12)
C(18)-C(19)	1.413(13)
C(19A)-C(20A)	1.375(13)
C(4A)-C(5A)	1.372(14)
C(4A)-C(3A)	1.403(14)
C(13)-C(12)	1.408(13)
C(8A)-C(13A)	1.340(14)
C(1A)-C(6A)	1.365(14)
C(1A)-C(2A)	1.389(14)
C(24)-C(28)	1.517(12)
C(24)-C(23)	1.523(12)
C(24)-C(27)	1.540(14)
C(13A)-C(12A)	1.411(12)
C(2A)-C(3A)	1.365(13)
C(6)-C(5)	1.389(12)
C(19)-C(20)	1.387(14)
C(20A)-C(21A)	1.384(15)
C(3)-C(2)	1.378(13)
C(3)-C(4)	1.400(15)
C(20)-C(21)	1.363(16)
C(5A)-C(6A)	1.378(13)
C(5)-C(4)	1.363(14)

Table S 5. Values of selected angles for FL-BTD-Ar and FL-BTD-OAr.

**FL-BTD-Ar**		**FL-BTD-OAr**	
**Atoms**	**Angle**	**Atoms**	**Angle**
N(2)-S-N(1)	101.91(5)	N(3)-S(1)-N(2)	101.6(4)
C(1)-O-C(7)	117.03(10)	N(3A)-S(1A)-N(2A)	100.6(4)
C(9)-N(1)-S	105.85(7)	C(8A)-O(2A)-C(1A)	117.3(7)
C(10)-N(2)-S	105.72(7)	C(4A)-O(1A)-C(7A)	118.9(10)
N(1)-C(9)-C(8)	125.38(9)	C(4)-O(1)-C(7)	119.0(9)
N(1)-C(9)-C(10)	113.11(9)	C(10)-N(3)-S(1)	107.7(6)
C(8)-C(9)-C(10)	121.49(9)	C(10A)-N(3A)-S(1A)	107.0(5)
N(2)-C(10)-C(11)	125.32(9)	C(8)-O(2)-C(1)	117.5(7)
N(2)-C(10)-C(9)	113.39(9)	C(9A)-N(2A)-S(1A)	105.5(6)
C(11)-C(10)-C(9)	121.29(9)	C(26A)-C(14A)-C(15A)	117.2(7)
C(13)-C(8)-C(9)	115.48(9)	C(26A)-C(14A)-C(11A)	123.5(7)
C(13)-C(8)-C(4)	122.97(9)	C(15A)-C(14A)-C(11A)	119.2(7)
C(9)-C(8)-C(4)	121.54(9)	C(22A)-C(23A)-C(18A)	120.5(8)
C(17)-C(18)-C(26)	120.87(9)	C(22A)-C(23A)-C(24A)	128.9(8)
C(17)-C(18)-C(19)	130.46(9)	C(18A)-C(23A)-C(24A)	110.6(7)
C(26)-C(18)-C(19)	108.67(9)	C(9)-N(2)-S(1)	104.2(6)
C(5)-C(4)-C(3)	118.25(9)	C(26)-C(25)-C(17)	120.7(7)
C(5)-C(4)-C(8)	120.99(9)	C(26)-C(25)-C(24)	129.1(7)
C(3)-C(4)-C(8)	120.75(9)	C(17)-C(25)-C(24)	110.2(7)
C(15)-C(17)-C(18)	118.83(9)	C(25)-C(26)-C(14)	121.1(8)
C(11)-C(12)-C(13)	123.17(9)	C(15)-C(14)-C(26)	116.6(8)
C(27)-C(26)-C(18)	120.04(9)	C(15)-C(14)-C(11)	120.4(7)
C(27)-C(26)-C(25)	129.19(9)	C(26)-C(14)-C(11)	123.0(8)
C(18)-C(26)-C(25)	110.76(9)	N(3)-C(10)-C(9)	110.6(7)
C(15)-C(14)-C(27)	119.46(9)	N(3)-C(10)-C(11)	127.3(7)
C(15)-C(14)-C(11)	120.10(9)	C(9)-C(10)-C(11)	122.1(7)
C(27)-C(14)-C(11)	120.43(9)	C(25A)-C(24A)-C(23A)	101.0(6)
C(26)-C(27)-C(14)	119.63(9)	C(25A)-C(24A)-C(27A)	113.2(7)
C(17)-C(15)-C(14)	121.06(9)	C(23A)-C(24A)-C(27A)	111.2(7)
C(20)-C(19)-C(24)	121.25(10)	C(25A)-C(24A)-C(28A)	112.1(7)
C(20)-C(19)-C(18)	130.63(10)	C(23A)-C(24A)-C(28A)	110.3(7)

C(24)-C(19)-C(18)	108.07(9)
C(2)-C(3)-C(4)	120.87(9)
C(12)-C(11)-C(10)	115.57(9)
C(12)-C(11)-C(14)	122.74(9)
C(10)-C(11)-C(14)	121.69(9)
C(8)-C(13)-C(12)	122.99(9)
C(5)-C(6)-C(1)	119.59(9)
C(24)-C(25)-C(26)	100.98(8)
C(24)-C(25)-C(29)	112.15(9)
C(26)-C(25)-C(29)	113.09(8)
C(24)-C(25)-C(28)	111.17(9)
C(26)-C(25)-C(28)	110.16(8)
C(29)-C(25)-C(28)	109.11(8)
C(3)-C(2)-C(1)	120.09(10)
C(6)-C(5)-C(4)	121.24(9)
O-C(1)-C(6)	124.49(10)
O-C(1)-C(2)	115.57(9)
C(6)-C(1)-C(2)	119.93(10)
C(23)-C(24)-C(19)	119.73(10)
C(23)-C(24)-C(25)	129.00(10)
C(19)-C(24)-C(25)	111.27(9)
C(21)-C(20)-C(19)	118.54(11)
C(24)-C(23)-C(22)	118.99(11)
C(20)-C(21)-C(22)	120.39(12)
C(21)-C(22)-C(23)	121.11(12)
C(27A)-C(24A)-C(28A)	108.9(7)
C(17)-C(16)-C(15)	118.9(8)
C(12A)-C(11A)-C(10A)	113.2(7)
C(12A)-C(11A)-C(14A)	123.2(8)
C(10A)-C(11A)-C(14A)	123.6(7)
C(25A)-C(26A)-C(14A)	119.7(8)
N(3A)-C(10A)-C(9A)	111.6(7)
N(3A)-C(10A)-C(11A)	126.0(7)
C(9A)-C(10A)-C(11A)	122.3(7)
C(23A)-C(18A)-C(19A)	120.8(8)
C(23A)-C(18A)-C(17A)	109.6(7)
C(19A)-C(18A)-C(17A)	129.5(8)
C(13)-C(8)-O(2)	128.4(8)
C(13)-C(8)-C(9)	116.0(7)
O(2)-C(8)-C(9)	115.6(8)
C(16A)-C(17A)-C(25A)	119.8(7)
C(16A)-C(17A)-C(18A)	132.5(8)
C(25A)-C(17A)-C(18A)	107.6(7)
N(2)-C(9)-C(10)	115.8(7)
N(2)-C(9)-C(8)	123.2(8)
C(10)-C(9)-C(8)	121.0(8)
C(21)-C(22)-C(23)	118.7(9)
C(23A)-C(22A)-C(21A)	119.0(9)
C(16)-C(17)-C(25)	119.9(8)
C(16)-C(17)-C(18)	130.9(8)
C(25)-C(17)-C(18)	109.2(7)
C(16A)-C(15A)-C(14A)	123.1(8)
C(26A)-C(25A)-C(17A)	121.4(7)
C(26A)-C(25A)-C(24A)	127.6(8)
C(17A)-C(25A)-C(24A)	111.0(7)
C(12)-C(11)-C(10)	113.4(8)
C(12)-C(11)-C(14)	122.6(8)
C(10)-C(11)-C(14)	123.9(7)
N(2A)-C(9A)-C(10A)	115.2(7)

N(2A)-C(9A)-C(8A)	124.6(7)
C(10A)-C(9A)-C(8A)	120.2(8)
C(14)-C(15)-C(16)	122.6(7)
C(6)-C(1)-C(2)	121.6(8)
C(6)-C(1)-O(2)	119.0(9)
C(2)-C(1)-O(2)	119.2(9)
C(23)-C(18)-C(19)	120.4(8)
C(23)-C(18)-C(17)	108.3(7)
C(19)-C(18)-C(17)	131.2(8)
C(17A)-C(16A)-C(15A)	118.5(8)
C(20A)-C(19A)-C(18A)	117.9(9)
O(1A)-C(4A)-C(5A)	116.2(9)
O(1A)-C(4A)-C(3A)	124.5(9)
C(5A)-C(4A)-C(3A)	119.2(8)
C(8)-C(13)-C(12)	122.2(8)
C(13A)-C(8A)-O(2A)	127.9(8)
C(13A)-C(8A)-C(9A)	116.6(8)
O(2A)-C(8A)-C(9A)	115.5(8)
C(11)-C(12)-C(13)	125.3(9)
C(6A)-C(1A)-O(2A)	119.9(9)
C(6A)-C(1A)-C(2A)	119.5(8)
O(2A)-C(1A)-C(2A)	120.4(9)
C(28)-C(24)-C(23)	112.2(8)
C(28)-C(24)-C(25)	112.7(7)
C(23)-C(24)-C(25)	101.0(7)
C(28)-C(24)-C(27)	109.6(8)
C(23)-C(24)-C(27)	110.5(8)
C(25)-C(24)-C(27)	110.5(7)
C(8A)-C(13A)-C(12A)	122.7(8)
C(3A)-C(2A)-C(1A)	120.4(10)
C(1)-C(6)-C(5)	117.9(9)
C(22)-C(23)-C(18)	120.0(8)
C(22)-C(23)-C(24)	128.8(8)
C(18)-C(23)-C(24)	111.2(7)

C(20)-C(19)-C(18)	117.8(9)
C(19A)-C(20A)-C(21A)	121.6(9)
C(11A)-C(12A)-C(13A)	125.0(8)
C(22A)-C(21A)-C(20A)	120.2(9)
C(2)-C(3)-C(4)	119.1(9)
C(21)-C(20)-C(19)	121.8(10)
C(4A)-C(5A)-C(6A)	120.2(9)
C(4)-C(5)-C(6)	122.3(10)
C(20)-C(21)-C(22)	121.3(9)
O(1)-C(4)-C(5)	116.6(9)
O(1)-C(4)-C(3)	124.8(9)
C(5)-C(4)-C(3)	118.6(8)
C(1)-C(2)-C(3)	120.5(9)
C(2A)-C(3A)-C(4A)	119.8(10)
C(1A)-C(6A)-C(5A)	120.7(9)

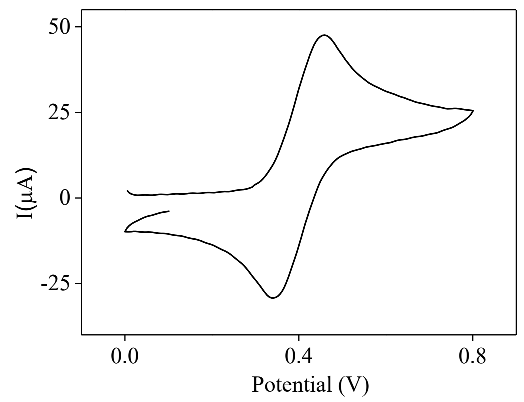

Figure S 4. Cyclic voltammetry curves of ferrocene.

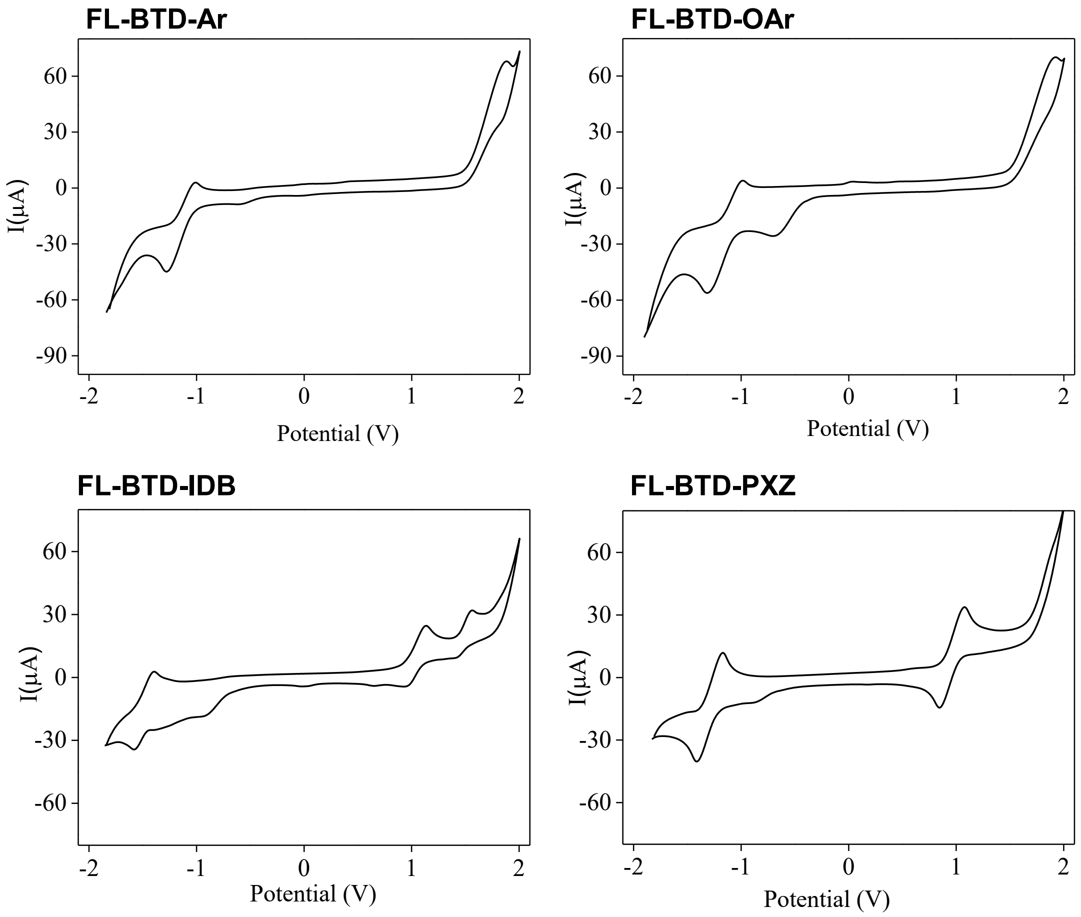

Figure S 5. Cyclic voltammetry curves of FL-BTD derivatives.

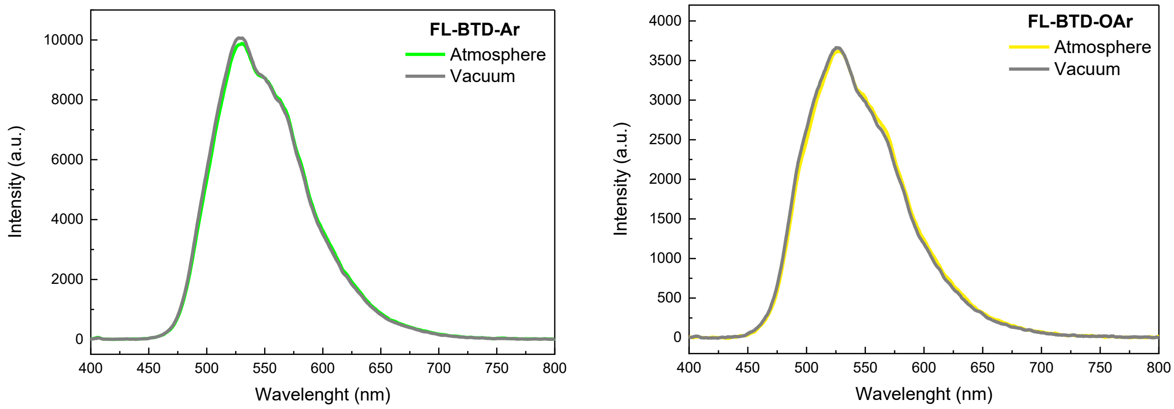

Figure S 6. PL spectrum with atmospheric air and under vacuum in films.

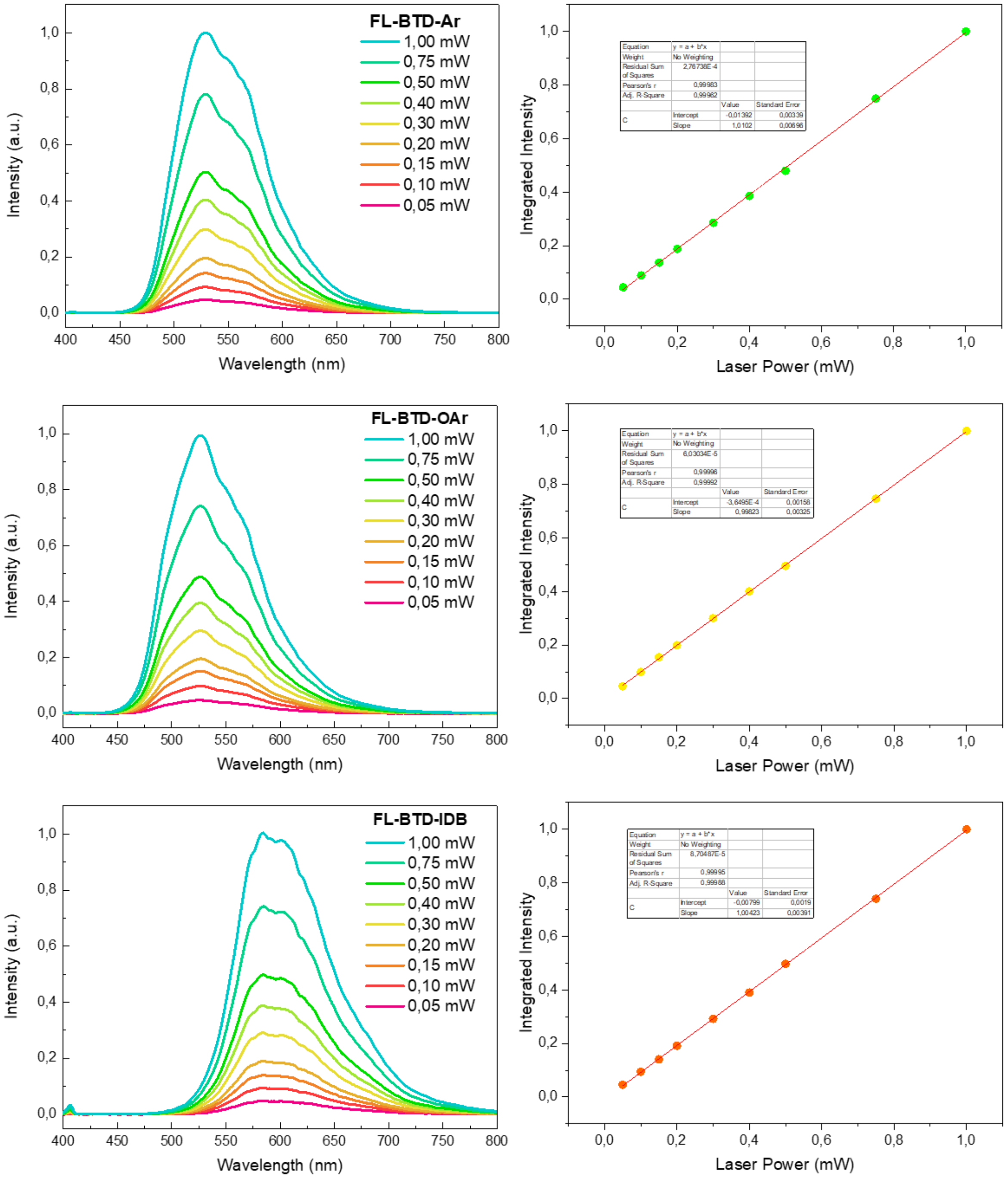

Figure S 7. PL emission of FL-BTD derivatives in films excited at different laser pulse power (left) and their emission intensity dependence for laser pulse power at room temperature (right).

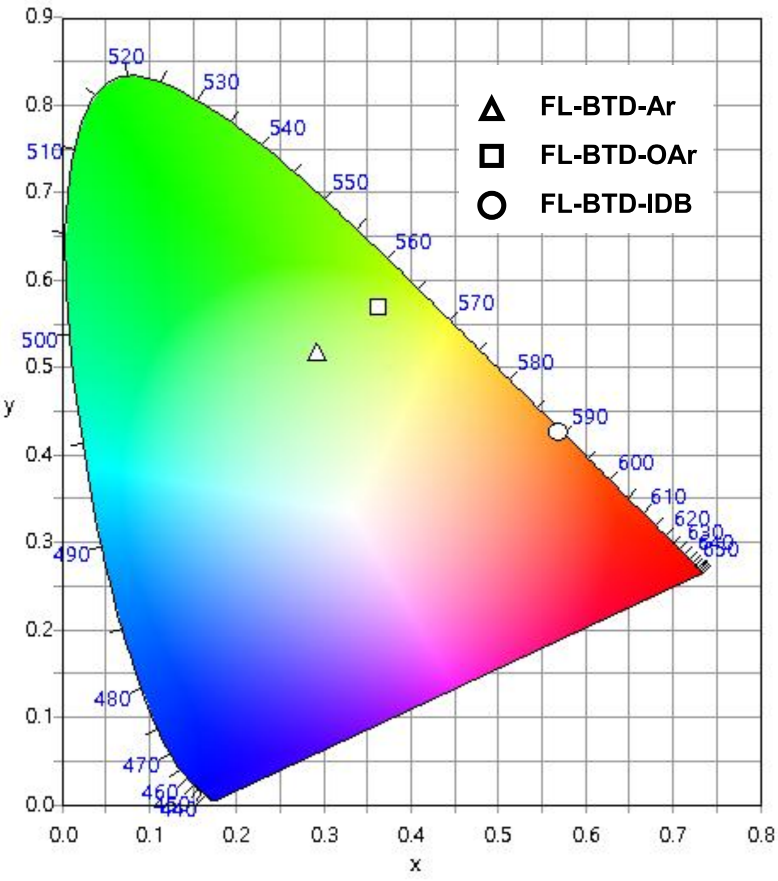

Figure S 8. CIE coordinates for OLEDs using FL-BTD derivatives as emitting layers.

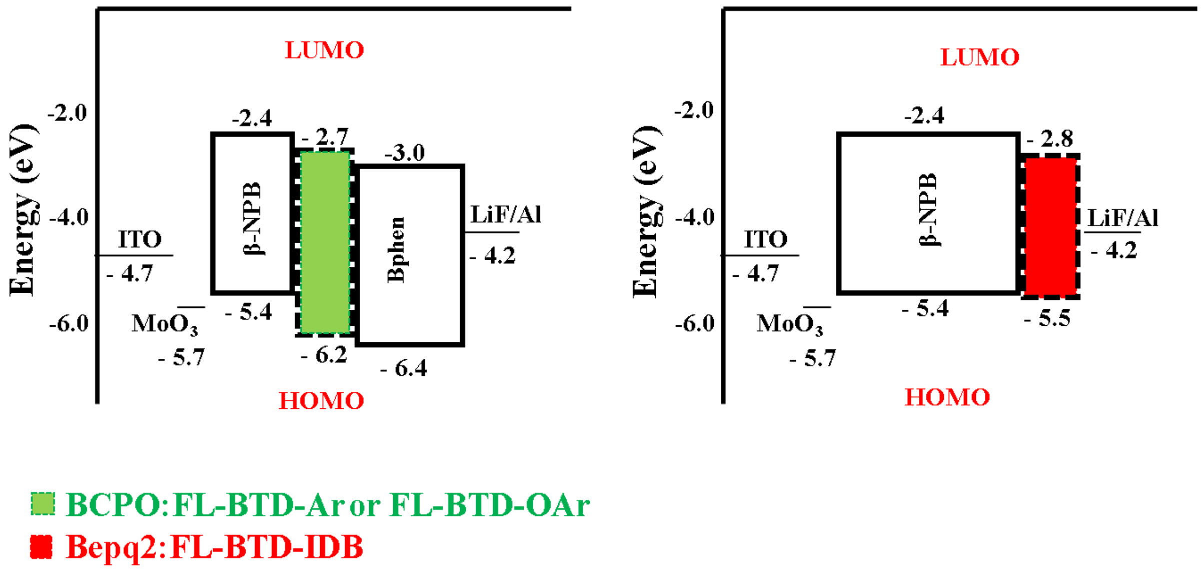

Figure S 9. Energy-level diagram of FL-BTD derivative based OLEDs.

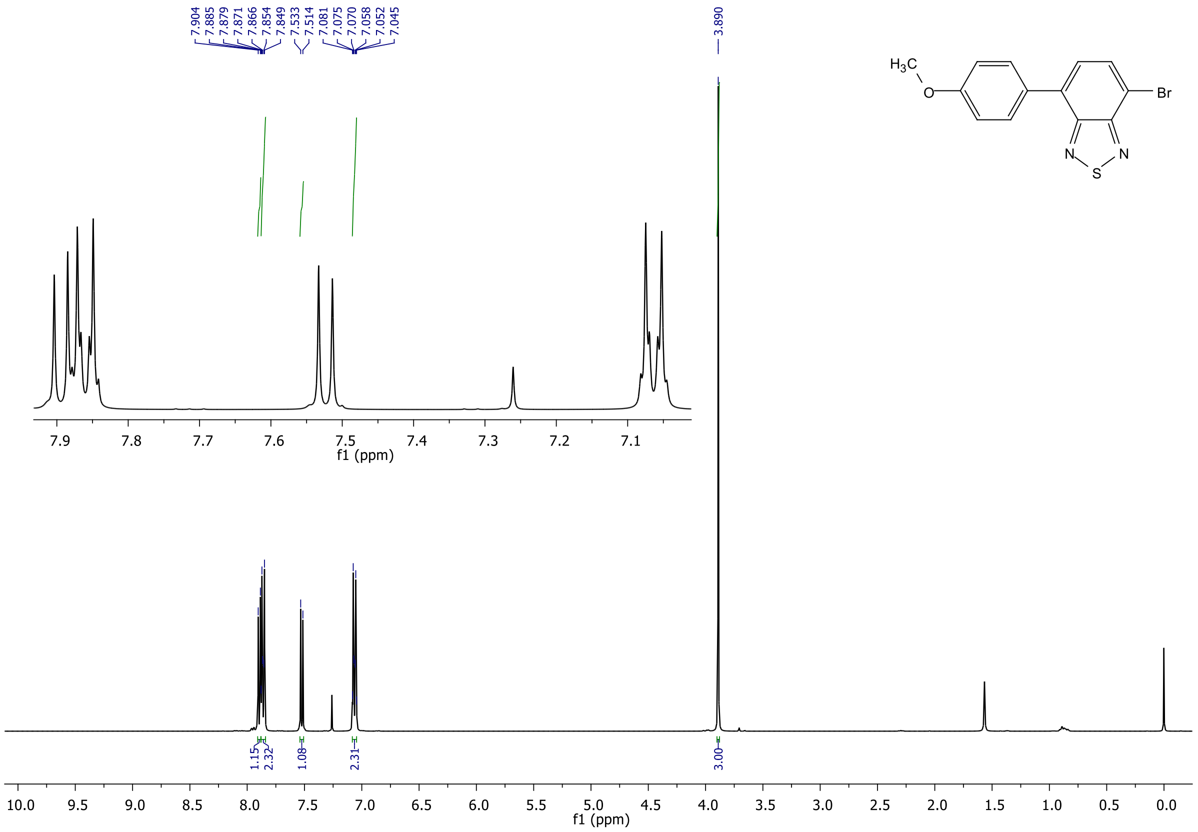

Figure S 10. ^{1}H NMR spectrum of 2 (400 MHz, $CDCl_3$).

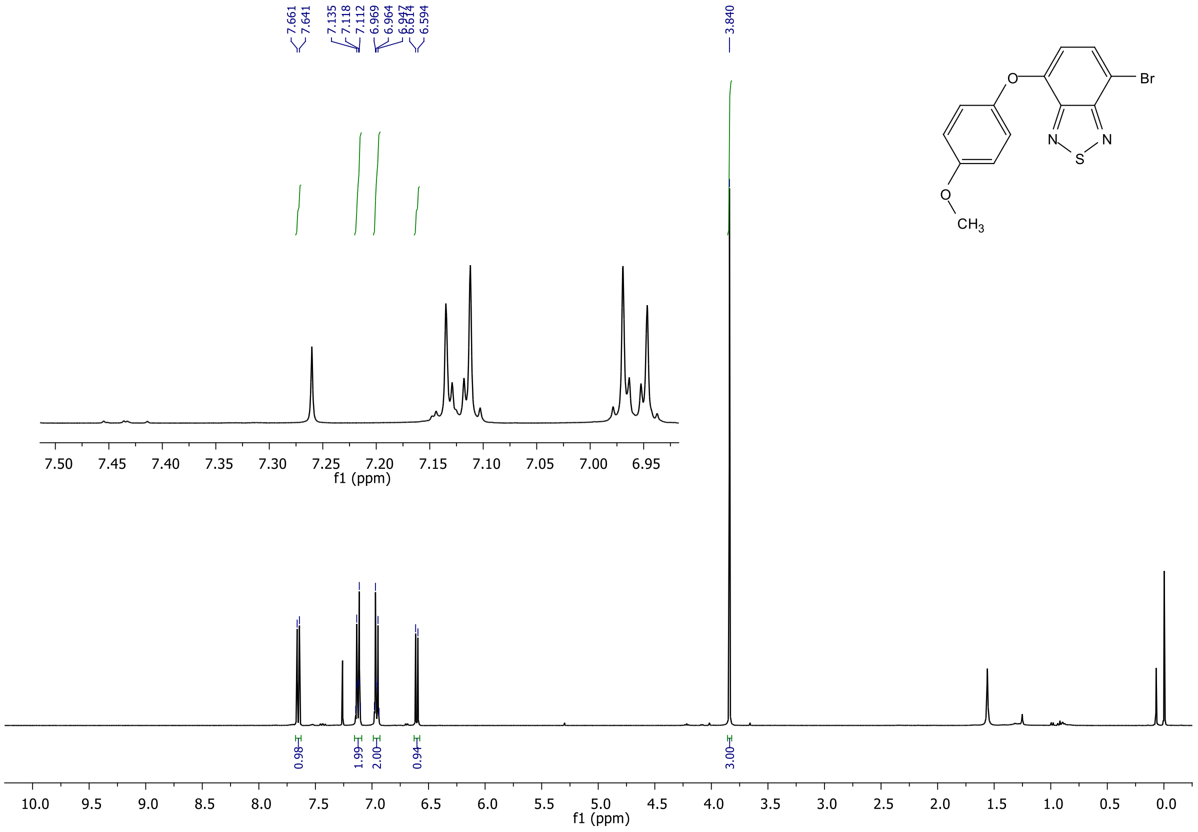

Figure S 11. ^{1}H NMR spectrum of 3 (400 MHz, $CDCl_3$).

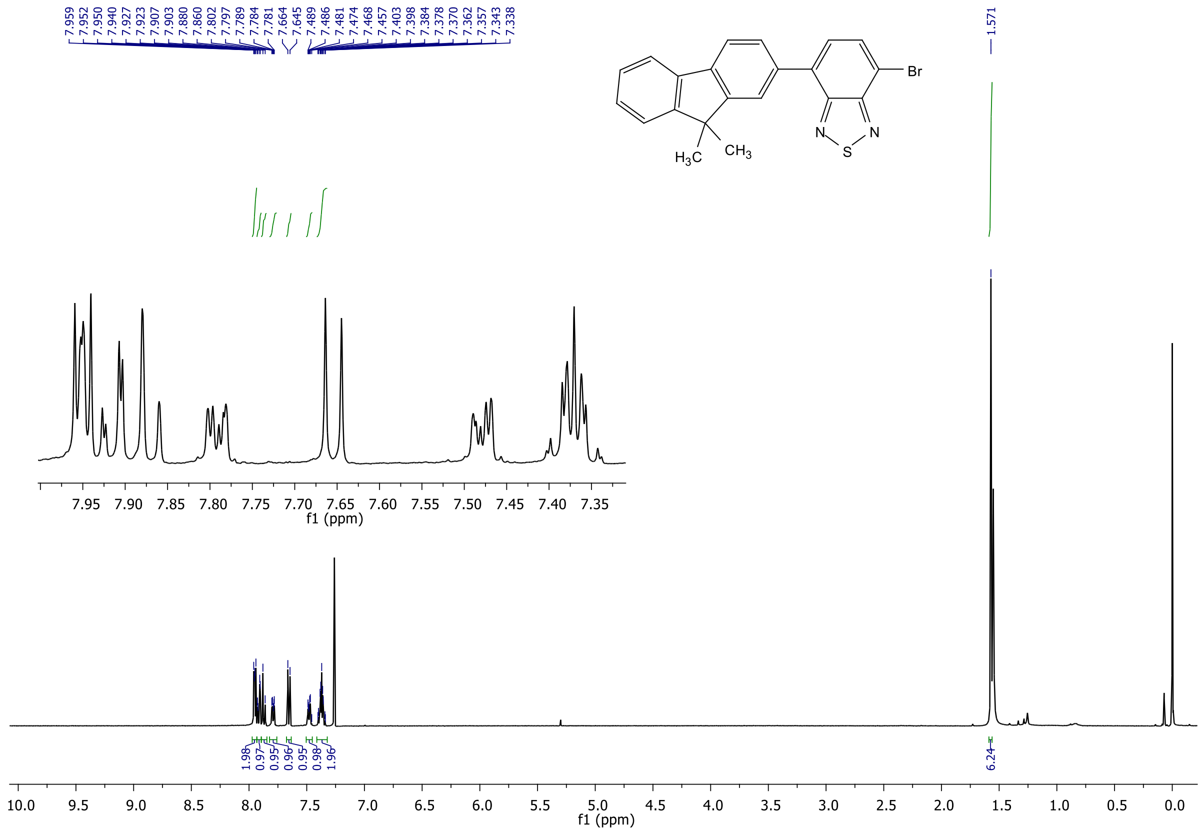

Figure S 12. ^{1}H NMR spectrum of 4 (400 MHz, $CDCl_3$).

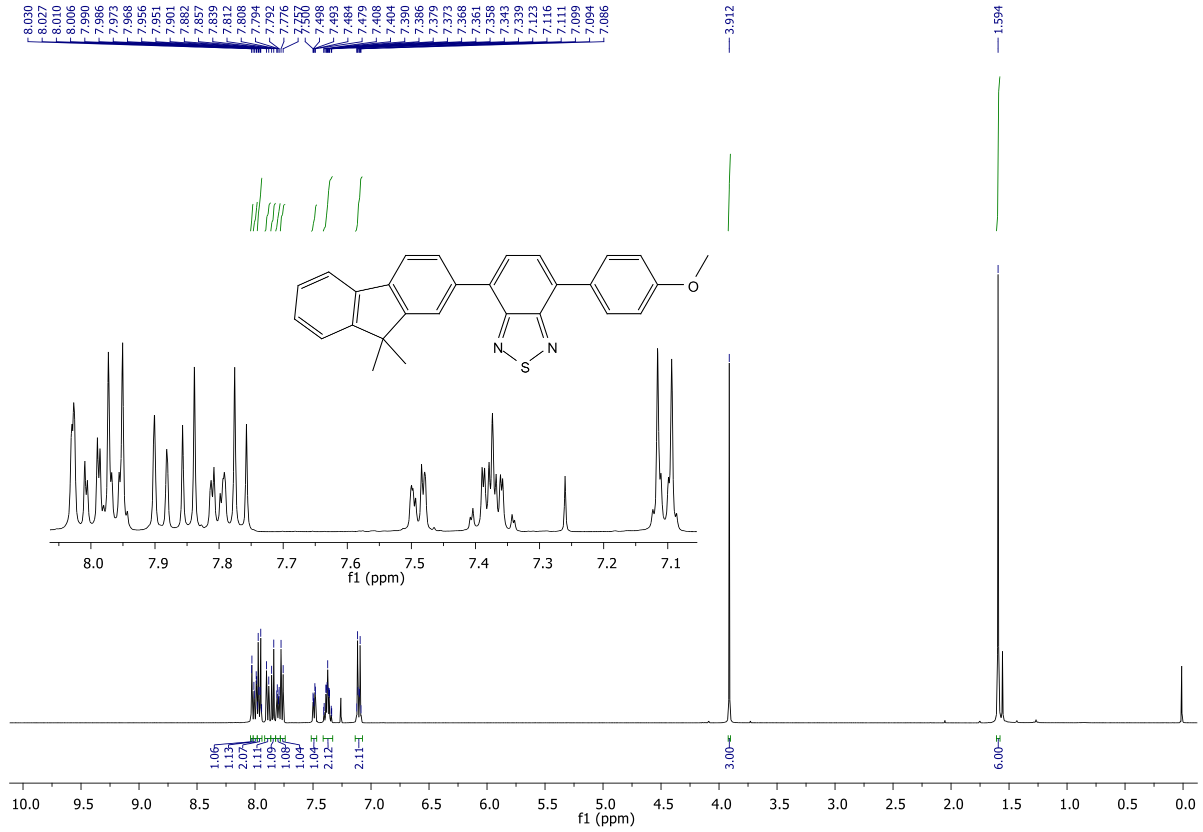

Figure S 13. ^{1}H NMR spectrum of FL-BTD-Ar (400 MHz, $CDCl_3$).

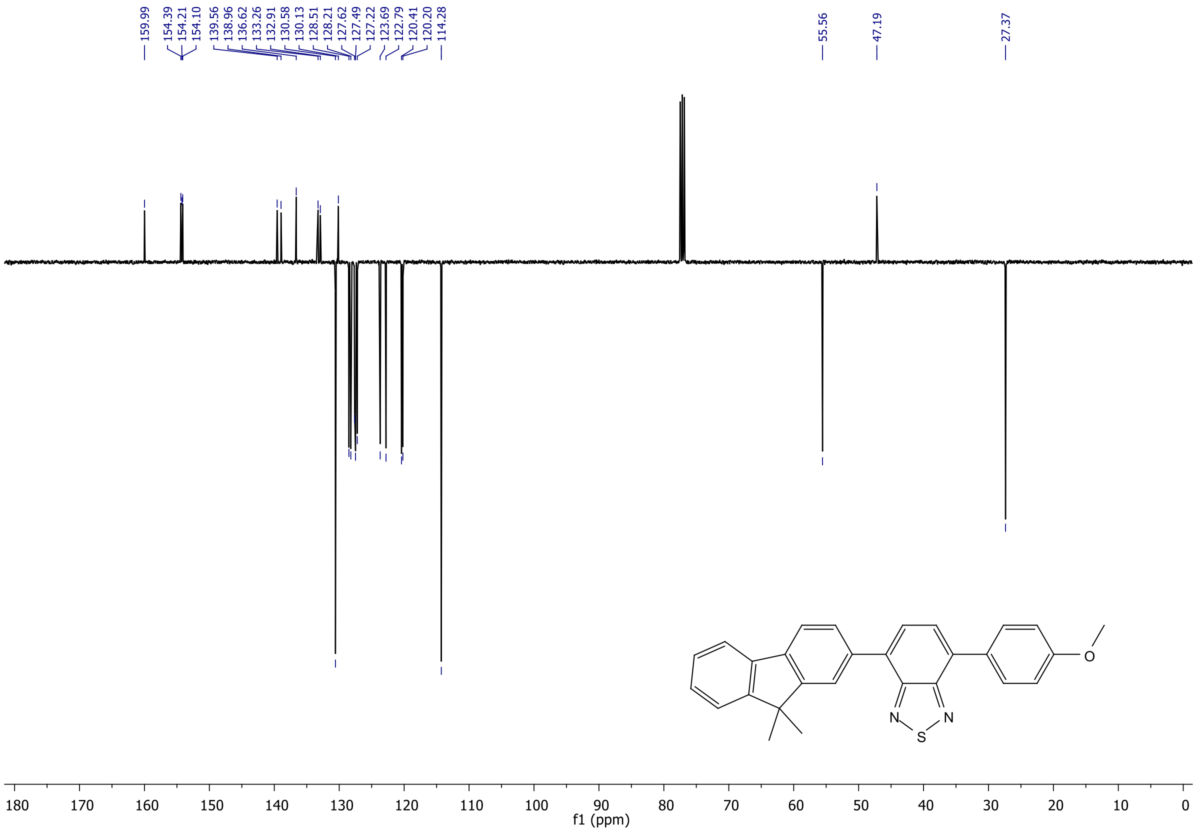

Figure S 14. ^{13}C NMR (APT) spectrum of **FL-BTD-Ar** (101 MHz, CDCl$_3$).

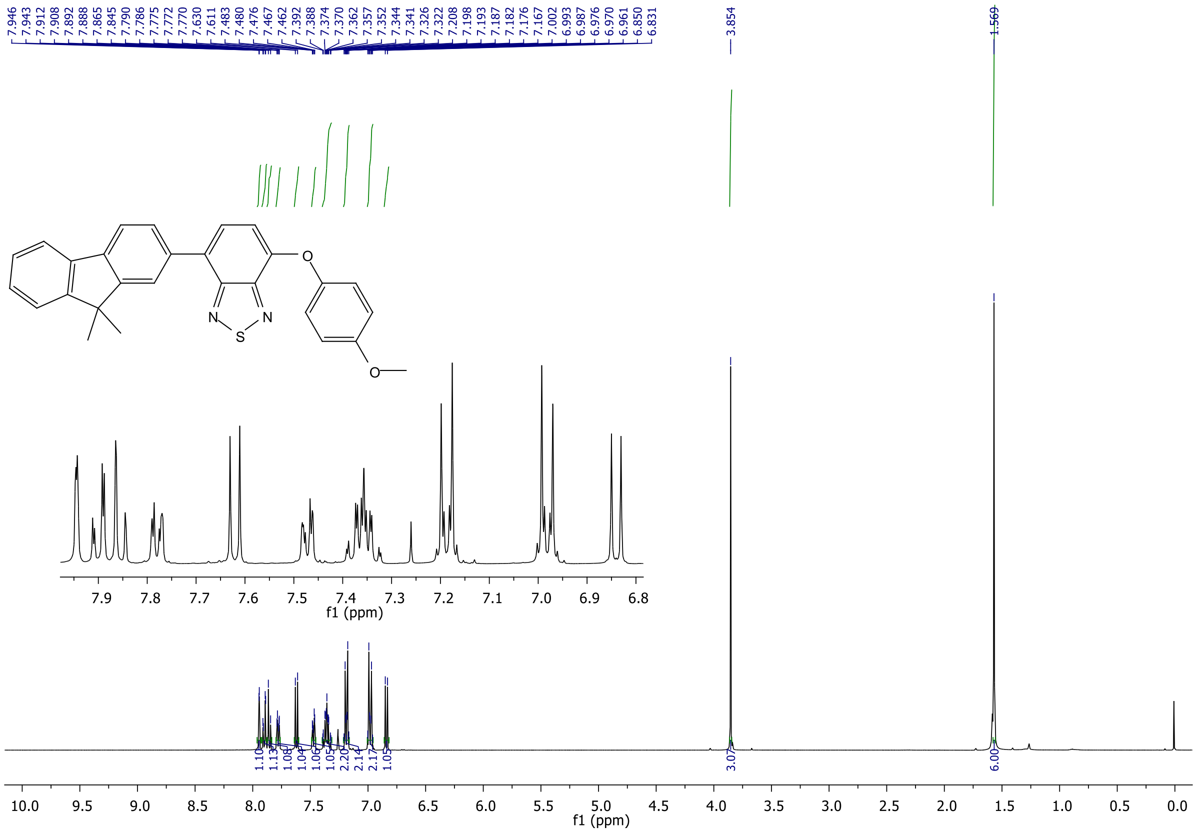

Figure S 15. ^{1}H NMR spectrum of **FL-BTD-OAr** (400 MHz, CDCl$_3$).

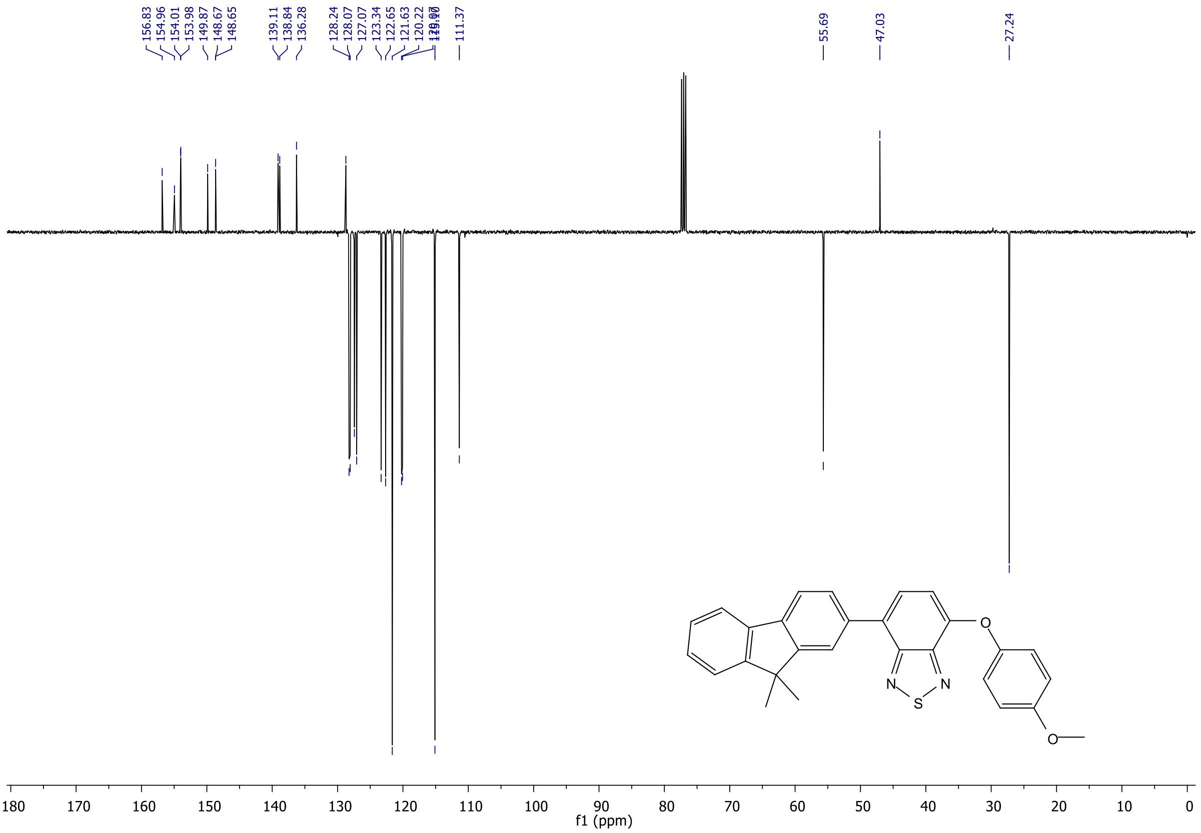

Figure S 16. ^{13}C NMR (APT) spectrum of **FL-BTD-OAr** (101 MHz, $CDCl_3$).

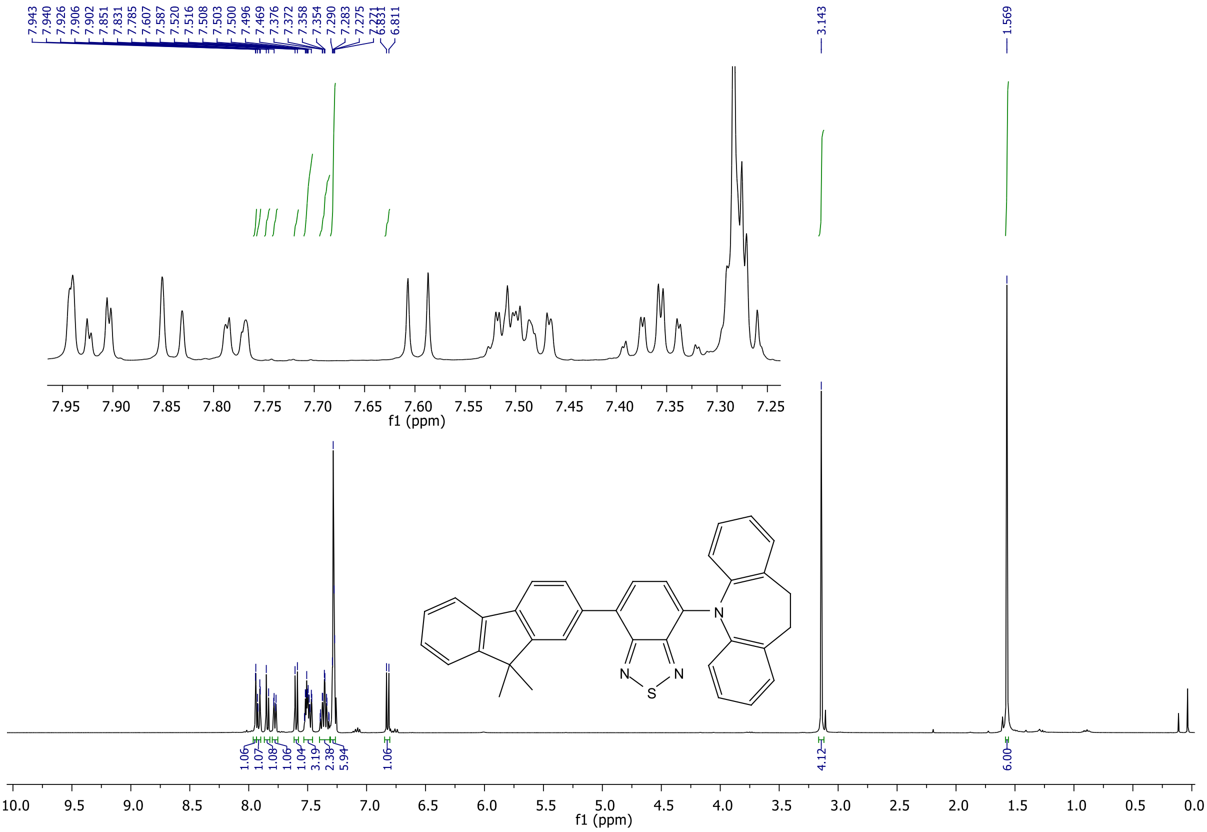

Figure S 17. ^{1}H NMR spectrum of **FL-BTD-IDB** (400 MHz, $CDCl_3$).

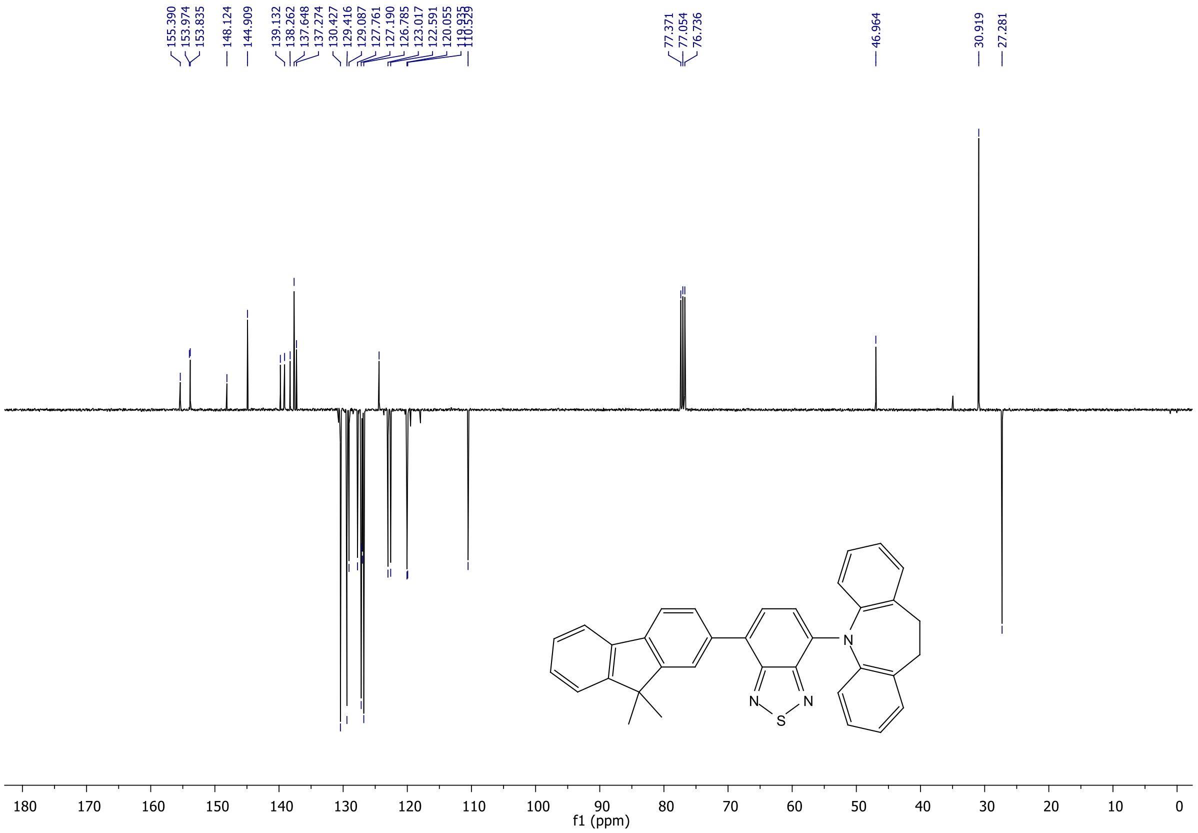

Figure S 18. ^{13}C NMR (APT) spectrum of **FL-BTD-IDB** (101 MHz, $CDCl_3$).

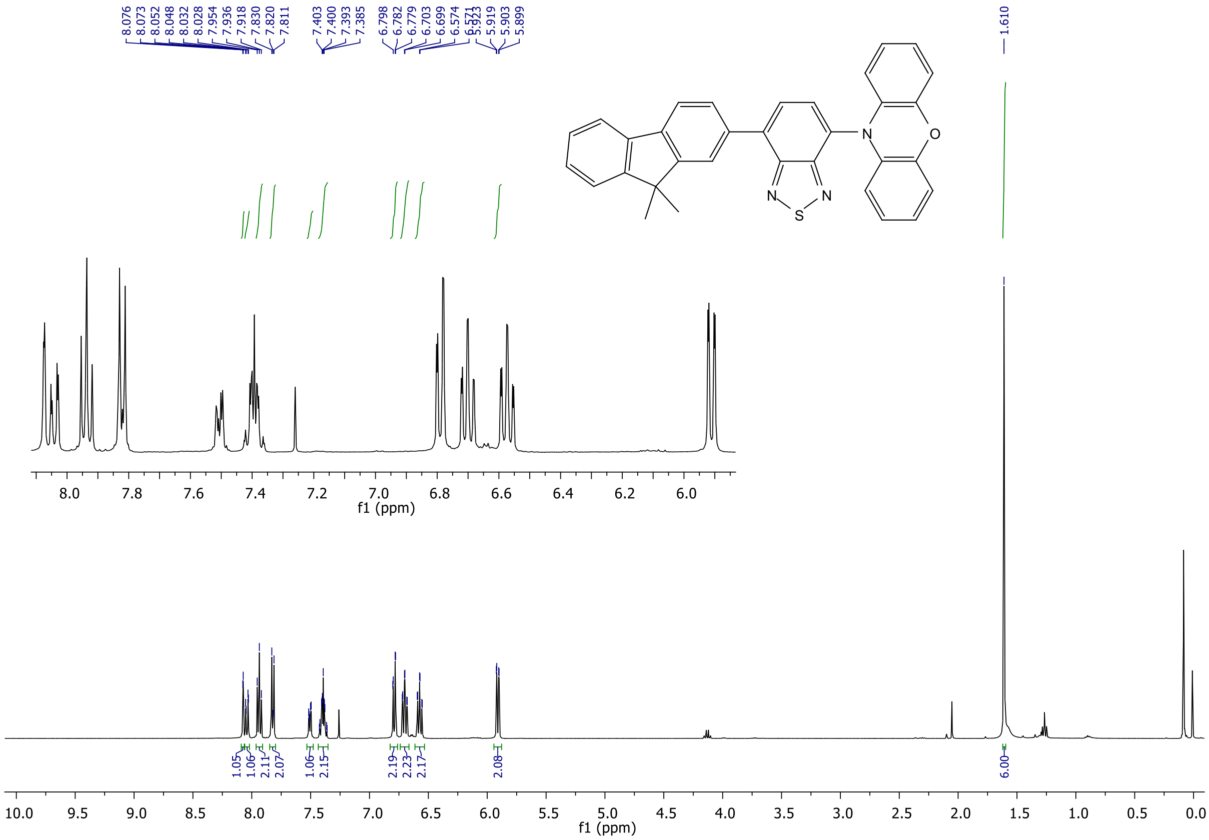

Figure S 19. ^{1}H NMR spectrum of **FL-BTD-PXZ** (400 MHz, $CDCl_3$).

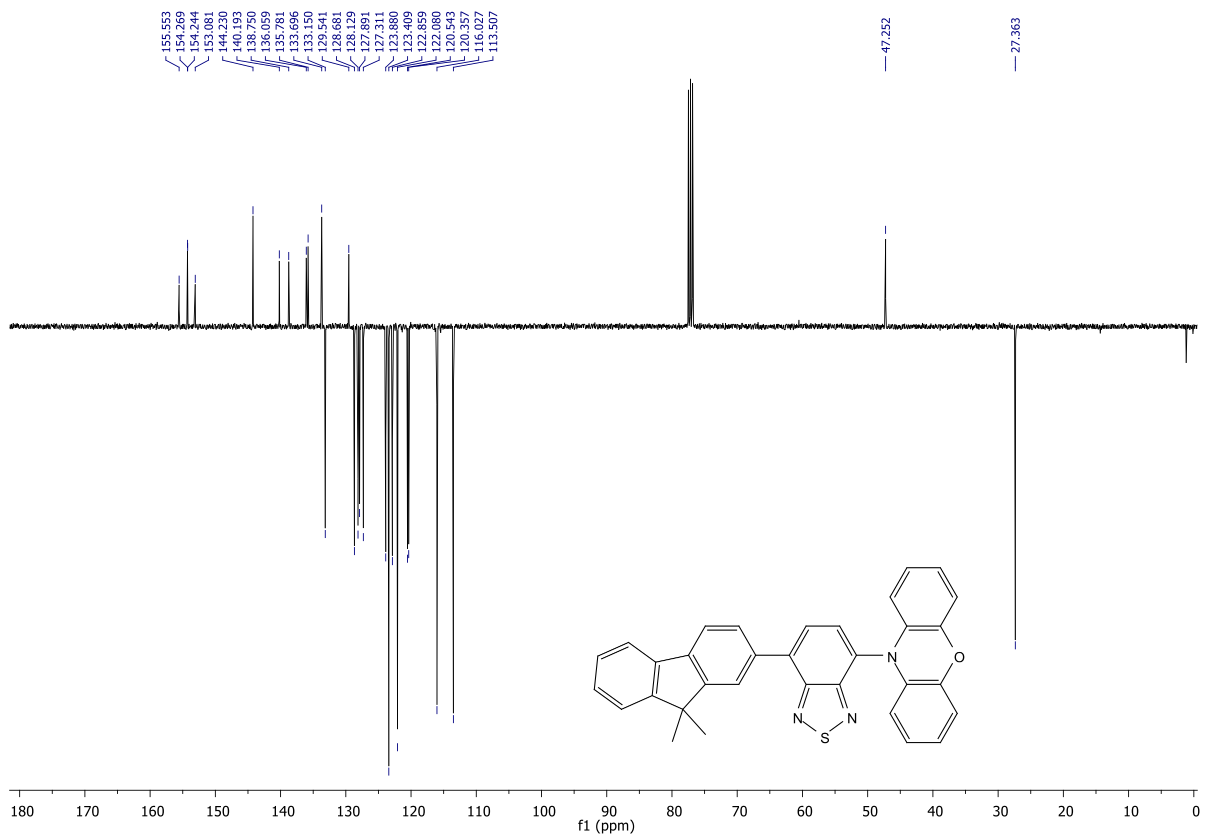

Figure S 20. ^{13}C NMR (APT) spectrum of **FL-BTD-PXZ** (101 MHz, $CDCl_3$).

Table S6. ^{1}H NMR assignment data for FL-BTD compounds, $CDCl_3$, 400 MHz.

		**FL-BTD-Ar**		
8.03	d	1.1	1	Position 1 of FL
8.00	dd	7.9, 1.6	1	Position 4 of FL
7.98-7.94	m		2	*m*- respect to methoxy in Ar
7.89	d	7.8	1	Position 3 of FL
7.85	d	7.3	1	Positions 5 and 6 of BTD
7.77	d	7.3	1	
7.82-7.78	m		1	
7.52-7.47	m		1	Positions 5-8 of FL
7.41-7.33	m		2	
7.14-7.07	m		2	*o*- respect to methoxy in Ar
3.91	s		3	CH_3 of methoxy group in Ar
1.59	s		6	Position 9 $(CH_3)_2$ of FL
		**FL-BTD-OAr**		
7.94	d	1.0	1	Position 1 of FL
7.90	dd	7.9, 1.6	1	Position 4 of FL
7.85	d	7.9	1	Position 3 of FL
7.62	d	7.9	1	Position 5 of BTD

7.80-7.76	m		1	
7.49-7.45	m		1	Positions 5-8 of FL
7.40-7.31	m		2	
7.21-7.16	m		2	*m*- respect to methoxy in OAr
7.01-6.96	m		2	*o*- respect to methoxy in OAr
6.84	d	7.9	1	Position 6 of BTD
3.85	s		3	CH_3 of methoxy group in OAr
1.57	s		6	Position 9 $(CH_3)_2$ of FL
		**FL-BTD-IDB**		
7.94	d	1.1	1	Position 1 of FL
7.91	dd	7.9, 1.5	1	Position 4 of FL
7.84	d	7.9	1	Position 3 of FL
7.60	d	8.0	1	Position 5 of BTD
7.78	dd	6.5, 1.5	1	
7.53-7.46	m		3	Aromatic Hs in IDB and FL
7.40-7.31	m		2	
7.31-7.26	m		6	
6.82	d	8.0	1	Position 6 of BTD
3.14	s		4	$(CH_2)_2$ in IDB
1.57	s		6	Position 9 $(CH_3)_2$ of FL
		**FL-BTD-PXZ**		
8.07	d	1.3	1	Position 1 of FL
8.04	dd	7.9, 1.5	1	Position 4 of FL
7.96-7.91	m		2	
7.85-7.80	m		2	Aromatic Hs in BTD and FL
7.53-7.48	m		1	
7.44-7.35	m		2	
6.79	dd	7.9, 1.4	2	
6.70	td	7.7, 1.4	2	Aromatic Hs in PXZ
6.57	td	7.7, 1.5	2	
5.91	dd	7.9, 1.4	2	
1.61	s		6	Position 9 $(CH_3)_2$ of FL

Table S7. ^{13}C NMR assignment data for FL-BTD compounds (101 MHz, $CDCl_3$).

**Assignment**	**-Ar**	**-OAr**	**-IDB**	**-PXZ**
	**δ (ppm)**			
$(CH_3)_2$ of FL	27.4	27.2	27.3	27.4
C9 of FL	47.2	47.0	47.0	47.3
CHs of BTD	120.4<br>120.2	120.2<br>120.1	120.1<br>119.9	120.5<br>120.4
C=Ns of BTD	154.4<br>154.2	154.0<br>154.0	154.0<br>153.8,	154.3<br>154.2
CH_3 of methoxy group	55.6	55.7		
$(CH_2)_2$			30.9	

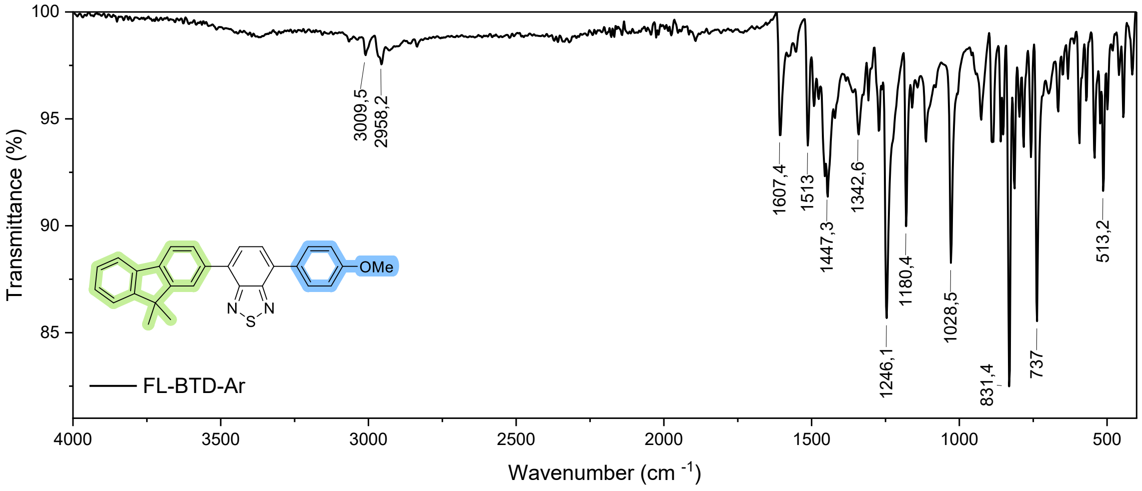

Figure S 21. FTIR (ATR) spectrum of **FL-BTD-Ar**.

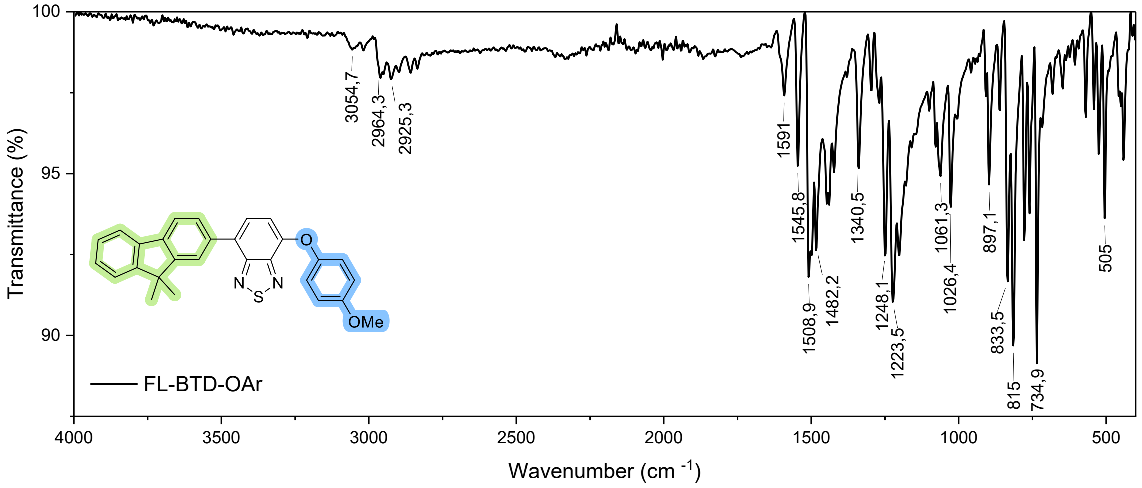

Figure S 22. FTIR (ATR) spectrum of **FL-BTD-OAr**.

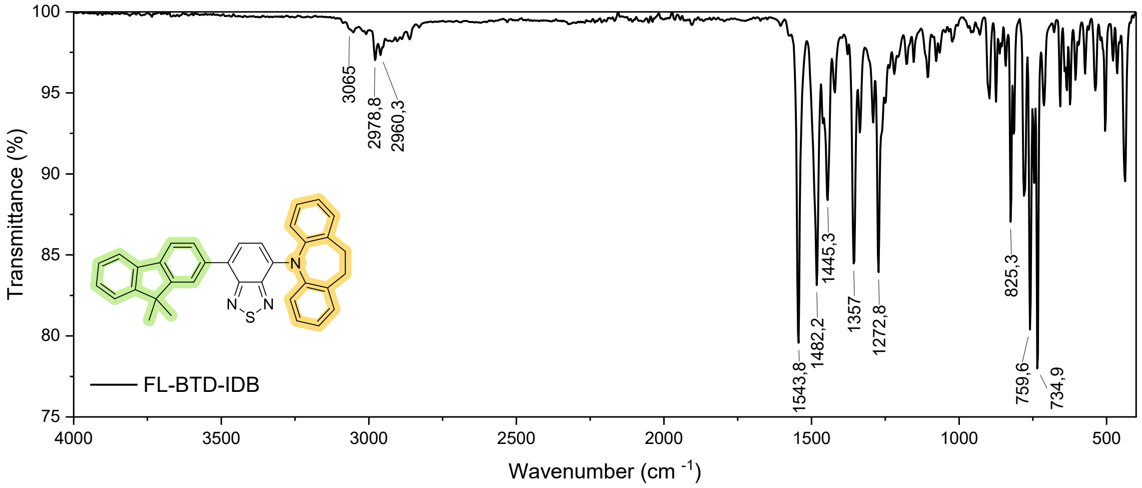

Figure S 23. FTIR (ATR) spectrum of **FL-BTD-IDB**.

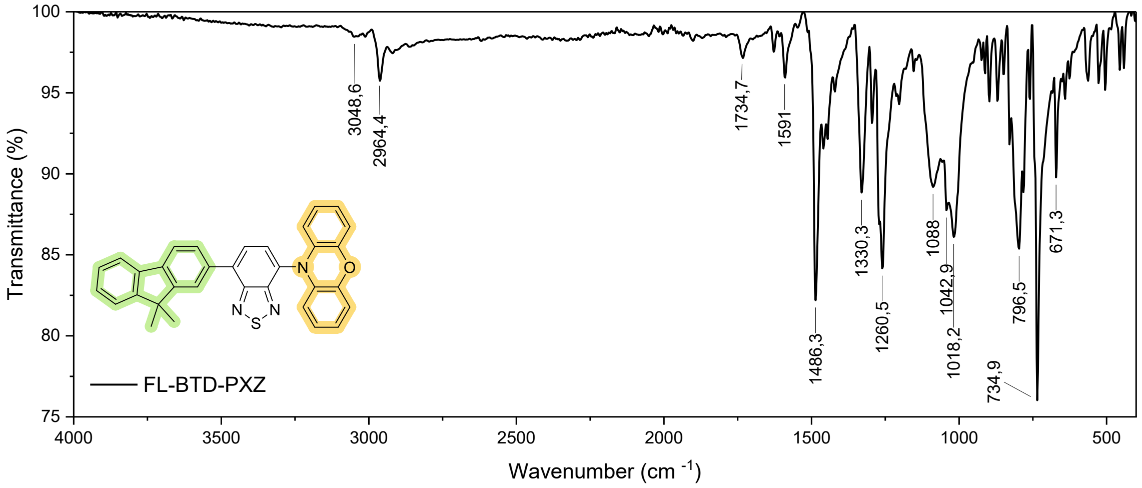

Figure S 24. FTIR (ATR) spectrum of **FL-BTD-PXZ**.
